# Links Between Feeding Preferences and Electroantennogram Response Profiles in Dung Beetles: The Importance of Dung Odor Bouquets

**DOI:** 10.1007/s10886-022-01383-1

**Published:** 2022-09-09

**Authors:** Miguel A. Urrutia, Vieyle Cortez, José R. Verdú

**Affiliations:** Research Institute CIBIO (Centro Iberoamericano de la Bioaffiliationersidad) Science Park, University of Alicante, E-03690 Alicante, Spain

**Keywords:** Trophic preference, Volatile organic compounds, Olfactometry, Electroantennography, Scarabaeoidea

## Abstract

The detection of dung odors is a crucial step in the food-searching behavior of dung beetles (Coleoptera: Scarabaeoidea). Yet, whether certain compounds characteristic of a given dung type contribute to a ‘choosy generalism’ behavior proposed for this taxonomic group is unknown. To address this, we analyzed the chemical composition of three types of dung (cow, horse, and rabbit) and conducted behavioral and electroantennogram (EAG) bioassays on 15 species of dung beetles using 19 volatile organic compounds representing the three dung samples. Chemical analyses revealed substantial qualitative and quantitative differences among dung types. When offered these food options in an olfactometer, 14 species exhibited a feeding preference. Surprisingly, all 19 compounds used in the EAG assays elicited antennal responses, with species displaying different olfactory profiles. The relationship between behavioral preferences and electrophysiological profiles highlighted that species with different food preferences had differences in antennal responses. Moreover, a specific set of EAG-active compounds (nonanal, sabinene, acetophenone, ρ-cresol, 2-heptanone, 1H-indole, and 6-methyl-5-hepten-2-one) were the strongest drivers in the distinct sensory profiles of the trophic preference groups. Our results point to the importance of the whole bouquet of dung-emanating compounds in driving food-searching behavior, but specific volatiles could aid in determining highly marked trophic preferences in certain species.

**Supplementary Information:**

The online version contains supplementary material available at 10.1007/s10886-022-01383-1.

## Introduction

A relevant distinction between animal consumers is whether they are specialized (monophagous and oligophagous) or generalized (polyphagous) in their diet. From a broad perspective, dung beetles could be classified as oligophagous if we consider that they are mainly coprophagous, a derived feeding habit associated with their filtering mouthparts adaptation, which arose from saprophagy in the Cretaceous, leading to a species radiation during mammal diversification in the Cenozoic (Ahrens et al. 2014; Gunter et al. 2016). However, some dung beetles *a priori* considered coprophagous, show a more generalist food selection, being able to exploit other types of resources, such as vertebrate and invertebrate carrion, fungi, and different fruits (Giménez Gómez et al. 2021; Halffter and Matthews 1966; Hanski and Cambefort 1991; Verdú et al. 2007; Weithmann et al. 2020). Within coprophagous species, due to the scattered and ephemeral presence of excrement, dung beetles show an opportunistic and generalized use of a wide range of dung types (Frank et al. 2018a; Hanski and Cambefort 1991), with species preferring only one dung type being very rare (Carpaneto et al. 2010; Galante and Cartagena 1999; Larsen et al. 2006; Lumaret and Iborra 1996). These observations are in congruence with the hypothesis of ‘choosy generalism’ proposed for coprophagous dung beetles (Dormont et al. 2004, 2007; Frank et al. 2018b).

Feces of different herbivorous mammals differ in terms of chemical and physical characteristics (Holter 2016). For example, the droppings of horses and cows, monogastric and ruminant herbivores respectively, differ in terms of their content of nutrients, plant fiber, water, and volatile compounds (Dormont et al. 2010; Frank et al. 2017, 2018b; Holter 2016; Stavert et al. 2014). At the extreme, certain excrement such as rabbit dung, which, due to its low nutritional and water content, is more similar to litter than to typical herbivore dung, e.g., horse or cow (Verdú and Galante 2004). This heterogeneity in chemical composition among different excrements of herbivorous mammals (Holter 2016; Nibaruta et al. 1980) could be a key factor in explaining the existence of feeding preferences in dung beetles.

Although there is evidence of preferences of dung beetles for certain dung types (Carpaneto et al. 2010; Dormont et al. 2004; Errouissi et al. 2004; Finn and Giller 2002; Frank et al. 2017; Giménez Gómez et al. 2021; Martín-Piera and Lobo 1996), there are few studies explaining the mechanisms that influence dung attraction through volatile organic compounds (VOCs). Dung beetles are attracted to dung by olfactory cues and their selection depends on the VOCs emitted by the different types of potential resource, the distance to the source, and the nutritional quality of each resource (Bogoni and Hernández 2014; Dormont et al. 2004, 2007, 2010; Hanski and Cambefort 1991; Holter and Scholtz 2007). Dung odors are complex mixtures, typically comprising between 50 and 400 VOCs (Aii et al. 1980; Amann et al. 2014; Dormont et al. 2010; Frank et al. 2018b). While some VOCs are common to all dung types, others appear to be characteristic of particular types (Dormont et al. 2010; Frank et al. 2018b). For example, common dung-emitted VOCs such as ρ-cresol, 1H-indole, and skatole (Dormont et al. 2007; Inouchi et al. 1988; Stavert et al. 2014) could serve as general cues for the presence of a food source. More specifically, ρ-cresol is an abundant VOC in cattle dung (Aii et al. 1980; Dormont et al. 2010; Frank et al. 2018b), several omnivore dung types (Blanes-Vidal et al. 2009; Stavert et al. 2014; Walton et al. 2013), as well as in a variety of domesticated and non-domesticated animal fecal samples (Apps et al. 2012; Martín et al. 2010; Terada et al. 1992). Skatole has been identified in various studies with pigs (Hobbs et al. 1996; Koziel et al. 2005), chickens (Cai et al. 2007), and various other vertebrates (Dehnhard et al. 1991). Dung beetles could use complex mixtures of VOCs as signals to detect and select preferred dung types by processing a few ‘key’ VOCs in odor (Stavert et al. 2014; Wurmitzer et al. 2017). Some studies suggest that dung beetles prefer complex mixtures rather than single compounds (Frank et al. 2018b; Wurmitzer et al. 2017). Dung beetles can be attracted in the field with a blend of VOCs, such as 2-butanone, butyric acid, 1H-indole, and skatole, or with butyric acid (Wurmitzer et al. 2017), or a blend of 1H-indole, skatole, phenol, butyric acid, 2-butanone, and ρ-cresol (Frank et al. 2018b), suggesting that mixtures are much more effective than single compounds alone. Despite information obtained in field and laboratory studies on attraction to certain mixtures of dung volatiles, electrophysiological studies on these compounds as possible semiochemicals are needed.

In insects, olfaction plays a key role in many aspects of life, including the search for food. The olfactory system of insects is remarkably sensitive, specific, and dynamic. Dung beetles have an acute olfactory sensitivity that allows them to locate ephemeral and often patchily distributed resources required for reproduction (Tribe and Burger 2011). Few studies have investigated the foraging behavior in dung beetles using electrophysiological techniques. Inouchi et al. (1988) studied the Japanese dung beetle, *Geotrupes auratus*, in which they demonstrated that single antennal olfactory cells were active to volatile compounds derived from dung, including 2-butanone, phenol, ρ-cresol, 1H-indole, and skatole. Likewise, in a study carried out with *Anoplotrupes stercorosus* using gas chromatography coupled with electroantennographic detection (GC–EAD), a large number of VOCs derived from carrion were active (Weithmann et al. 2020). Yet, the relationships between feeding preferences for a specific type of dung and the VOCs that elicit electrophysiological responses remain to be determined.

The objective of this study was to investigate, using a combination of behavioral and electrophysiological bioassays, whether specific VOCs characteristic of a type of dung may determine food preferences of dung beetle species. To do this, we first analyzed the characteristic VOCs of three types of excrement (cow, horse, and rabbit). Secondly, we analyzed the attraction and feeding preferences of a large number of dung beetle species, belonging to different taxonomic and functional groups, using olfactometer bioassays. Finally, we carried out electroantennogram (EAG) bioassays to determine VOCs that elicit responses in each species. We hypothesized that *choosy generalism* behavior proposed for coprophagous dung beetles may be related to the presence of a high number of VOCs that are physiologically active, and that the existence of food preferences might be explained by the presence of a few specific VOCs.

## Methods and Materials

*Dung Selection, Compositional Chemical Relations, and Candidate Semiochemicals.* We selected three dung types for this study: cow, horse, and rabbit. These types differ in content of nutrients, fiber, water, and VOCs (Dormont et al. 2010; Frank et al. 2017, 2018b; Goodrich et al. 1981; Holter 2016; Stavert et al. 2014; Verdú and Galante 2004). Furthermore, cows are ruminant animals, horses are monogastric and rabbits are monogastric that use cecotrophy to maximize nutrient intake from their food. Odor samples of cow and horse dung from different individuals (n = 3) were collected in the field at Picos de Europa National Park (Principado de Asturias, Spain). Odor samples were collected immediately after defecation to avoid insect colonization and physical/chemical alteration of the dung. For rabbit dung, fresh samples were collected at the Sierra de la Carrasqueta and at the surroundings of the University of Alicante (Alicante, Comunidad Valenciana, Spain), from different rabbit latrines and were brought to the laboratory in individual plastic freezer bags (Ziploc, SC Johnson & Son, Racine, WI).

Volatile emissions from dung types were sampled using headspace sorptive extraction (HSSE) with Twisters® (stir bar, 0.5mm thick, 10mm long, polydimethylsiloxane coating, Gerstel GmbH & Co. KG, Mülheim an der Ruhr, Germany). The Twisters® were cleaned per manufacturer recommendations with acetonitrile (HPLC-grade) and conditioned at 250°C for 15h with a flow of 75 ml.min^− 1^ purified helium. For HSSE, the Twister® was fixed within the headspace volume by magnetic force using a neodymium disc magnet (Ø 5mm, height 3mm) placed inside a glass chamber. The dung sample was covered with the headspace glass chamber and HSSE extraction carried out by static sampling. Twisters® were exposed to headspace for 1h at 22°C (ambient temperature) for cow and horse dung samples, while rabbit dung was sampled in an incubator at 37°C for 24h. Extraction time was established in previous assays. A total of three replicates were performed for each dung type. After extraction, each stir bar was removed with tweezers and placed in a 2 ml vial to be transported to the laboratory, where it was thermally desorbed in a gas chromatograph/mass spectrometer (GC/MS).

Analysis was carried out using a 6890 Agilent GC system coupled to a Agilent 5973 inert quadrupole MS equipped with a thermo desorption system (TDS2) and a cryo-focusing CIS-4 PTV injector (Gerstel). Thermal desorption used a Gerstel TDS 2 (Gerstel GmbH & Co. KG, Mülheim an der Ruhr, Germany) at 300°C for 10min, with a helium flow of 55 ml.min^− 1^. The GC was fitted with a DB-5 capillary column (30m x 0.25mm I.D., 0.25μm film thickness), and used helium as carrier gas with a constant flow of 1.4 ml.min^− 1^. The initial oven temperature was set at 60°C for 5min, and increased by 5°C.min^− 1^ to 250°C, then held for 10min. The injector, in split mode, and the MS transfer line were set at 250 and 280°C respectively. The MS quadrupole and source were set at 150 and 250°C respectively. Mass spectra were taken in EI mode at 70eV with a scan range of 40–450 *m/z* and a scanning rate of 2.65 scans/s.

GC/MS data were processed using MSD ChemStation software (Agilent Technologies Inc., Santa Clara, CA, USA). Tentative compound identification was done by comparison of mass spectra in the WILEY and NIST mass spectral libraries. We calculated retention indices of VOCs using an alkane standard mixture (C_7_-C_30_ dissolved in hexane; Sigma-Aldrich Chemie GmBh, Steinheim, Germany) applying the method of Van den Dool and Kratz (1963), and compared these against literature values (Adams, 2007). Identifications were confirmed by comparison of spectra and retention times with those of authentic standards when available. Commercial standards were obtained from chemical suppliers (Sigma-Aldrich Chemie GmBh, Steinheim, Germany), with ≥ 98% purity, and were run under the same conditions as samples. Identified compounds were expressed as percentage of the total content of compounds (relative abundance).

*Dung Beetle Species Selection.* A total of 15 species of dung beetles was collected in different localities in Spain and France (see Table1). To broaden the response range, species belonging to three families (Aphodiidae, Geotrupidae, and Scarabaeidae) from 12 different genera were selected. We collected these species searching in various types of dung, including cow and horse pats and rabbit latrines (Table1). Specimens were placed in aerated plastic containers (38 × 32 × 15cm) with moist towel paper. The containers were then placed in a cooler at 20°C until they arrived at the laboratory. Separate terrariums were prepared for each species and placed in a climate chamber at 15 ± 1°C with 65% relative humidity (RH) and a photoperiod of 14:10h (light: dark). To standardize the condition of beetles, only mature specimens were selected according to external age-grading methods such as abrasion of the fore tibiae in conjunction with cuticle hardness of the pronotum and elytra, which makes it possible to sort out individuals of approximately the same age (Tyndale-Biscoe 1984). This work conforms to the Spanish legal requirements including those relating to conservation and welfare.

**Table 1:** Dung beetle species collected for this study and results of dung type most preferred by species according to olfactometry bioassays

Species	Location	Date		Dung collected in	Diel activity^2^	Dung preference group^3^
Aphodiidae						
*Ammoecius elevatus* (Olivier, 1879)	Cañada de los Potros, Sierra Nevada National Park, Andalusia, Spain.	August 2020		Cow (semidry)^1^	Diurnal	Cow
*Anomius baeticus* (Mulsant and Rey, 1869)	Cañada de los Potros, Sierra Nevada National Park, Andalusia, Spain.	August 2020		Cow (dry)^1^, rabbit	Crepuscular	Rabbit
*Aphodius fimetarius* (Linnaeus, 1758)	La Sauceda, Los Alcornocales Natural Park, Andalusia, Spain.	December 2020		Cow	Diurnal	Cow-Horse
Geotrupidae						
*Ceratophyus hoffmannseggi* (Fairmaire, 1856)	Doñana Biological Reserve, Doñana National Park, Andalusia, Spain.	November 2020		Horse	Nocturnal	Cow-Horse
*Jekelius hernandezi* (Lopez-Colon, 1988)	Corral Rubio, Albacete, Castilla la Mancha, Spain.	October 2020		Rabbit	Diurnal	Generalist
*Sericotrupes niger* (Marsham, 1802)	Cañada de los Potros, Sierra Nevada National Park, Andalusia, Spain.	August 2020		Cow	Crepuscular-nocturnal	Cow-Horse
*Thorectes valencianus* (Baraud, 1966)	Font Roja Natural Park, Alicante, Valencia, Spain.	May 2020		Rabbit	Diurnal	Rabbit
*Typhaeus typhoeus* (Linnaeus, 1758)	La Sauceda, Los Alcornocales Natural Park, Andalusia, Spain.	December 2020		Cow, horse	Crepuscular-nocturnal	Horse
Scarabaeidae						
*Ateuchetus cicatricosus* (Lucas, 1846)	Doñana Biological Reserve, Doñana National Park, Andalusia, Spain.	June 2020		Horse, cow	Diurnal	Cow
*Bubas bison* (Linnaeus, 1767)	Charco Redondo, Cadiz, Andalusia, Spain.	November 2020		Cow	Diurnal	Cow-Horse
*Copris hispanus* (Linnaeus, 1764)	Charco Redondo, Cadiz, Andalusia, Spain.	November 2020		Cow	Crepuscular-nocturnal	Horse
*Onthophagus emarginatus* (Mulsant & Godart, 1842)	Sierra de la Carrasqueta, Jijona, Valencia, Spain.	October 2020		Rabbit	Diurnal	Horse
*O. fracticornis* (Preyssler, 1790)	Les Angles, Pyrénées-Orientales, France.	August 2020		Cow	Diurnal	Cow-Horse
*O. maki* (Illiger, 1803)	Doñana Biological Reserve, Doñana National Park, Andalusia, Spain.	October 2020		Cow, horse	Diurnal	Cow
*O. melitaeus* (Fabricius, 1798)	La Sauceda, Los Alcornocales Natural Park, Andalusia, Spain.	November-December 2020		Cow, horse	Diurnal	Cow-Horse

^1^ Unless otherwise stated by the description of the physical state of the dung in parentheses, dung was freshly excreted

^2^ Diel activity describes the time of day when a species is most actively searching for food

^3^ Dung type most preferred according to olfactometry bioassays. For statistical results see Online Resource Fig. S1

*Food Preference using Olfactometer Tests.* Behavioral bioassays were carried out in 2020 to test food preferences of adult beetles to different dung types (cow, horse, and rabbit). Tests used a four-arm olfactometer design based on that of Verdú et al. (2007). We used two designs, adapted to the different sizes of dung beetles. For species over 1cm in length, the olfactometer consisted of a central circular arena (60cm superior diameter and 40cm inferior diameter) with four 5cm-diam. holes to attach tubes (arms) of methacrylate (50cm length, 5cm o.d., and 4.75cm i.d.) placed horizontally. For species less than 1cm in length, the central circular arena was reduced to 30 × 20cm (superior and inferior diameter, respectively), and the length of the methacrylate tubes was 30cm each. There was a plastic container with test samples at the end of each arm to capture beetles that responded positively to the tested resources. The plastic containers were designed to permit the entrance and exit of beetles that responded to the tested resources. Air was passed through an activated carbon filter and drawn into the plastic containers of the olfactometer. In the center of the arena was a hole in the methacrylate roof to attach a tube that conducted air to a fume hood. Complete sealing of the system was ensured by Teflon® to join all connections. Outside light was blocked off by wrapping the transparent pieces of the olfactometer with aluminum foil.

In each olfactometer, the source of VOCs consisted of three fresh dung samples (15g each) placed in the different containers, and an empty container for a control. The arena was covered with sterile vermiculite. After placing beetles in an arena, we waited 10min before starting an experiment, to allow beetles to adapt to the conditions. Each bioassay consisted of 3–22 replicates that were run on different days using a group of 20 beetles per session, except for *Ceratophyus hoffmannseggi* and *Ateuchetus cicatricosus* (10 beetles per bioassay). The bioassays were conducted at 28 ± 3°C during the day (08:00–16:00h) or night (20:00–08:00h) for diurnal and crepuscular-nocturnal species, respectively. After each trial, the olfactometer was disassembled, and its arena, tubes, and containers washed with neutral dishwashing soap (5%) and disinfected with 70% ethanol (v/v). We measured the number of individuals attracted to each dung type for each replicate. Beetles that made no choice after this time were considered a null response. In all cases, each beetle was tested only once and treatments were randomly interchanged.

*Electroantennography Bioassays.* For EAGs, characteristic VOCs of each dung type, as well as compounds shared between two or more dung types, were selected (see Statistical Analyses, for details). Synthetic compounds were > 95% pure and purchased from commercial sources (Sigma-Aldrich Chemie GmBh, Steinheim, Germany). For EAG recordings, each compound was diluted to 1% in hexane (HPLC-grade) and stored at -20°C until needed. Immediately prior to an experiment, 1µl of each test solution or hexane (control) was placed onto a filter paper strip (1 cm^2^, Whatman No. 1) and into a Pasteur pipette (15cm long), which served as the odor cartridge.

Signals were recorded with an EAG system (Syntech, Kirchzarten, Germany) consisting of a universal single-ended probe (Type PRG–2), a data acquisition interface board (Type IDAC–02), and a stimulus air-controller (CS–55). Antennae were excised from the heads of beetles using micro-scissors, inserted into small droplets of electrode gel (Spectra 360, Parker Laboratories, Fairfield, NJ, USA), and mounted individually between two metal electrodes in an antenna holder, under a purified air flow (500 ml.min^− 1^). A Syntech PC–based signal processing system was used to amplify and process EAG signals. Stimulation tests were carried out by applying a puff of humidified pure air (200 ml.min^− 1^) for 2s using a stimulus controller through an odor cartridge directed over the antenna through the main branch of a glass tube (7cm long × 5mm diam.). Testing began once a relatively stable baseline had been established. A control stimulus (hexane) was applied every five test stimuli. The signals were further analyzed using the EAG 2010 software (Syntech, Kirchzarten, Germany). Odorants were tested on 8–12 antennae from different individuals for each species. EAG responses were evaluated by measuring the maximum amplitude of polarity (mV) elicited by a stimulus. The absolute value of the EAG amplitude (mV) to each test stimulus was adjusted to compensate for the solvent (hexane) by subtracting the mean EAG response of the most recent control.

*Statistical Analyses.* To analyze the chemical profile differences between dung types, a Permutational Analysis of Variance (PERMANOVA) with Bray-Curtis dissimilarity (BCD) matrix was applied to the GC/MS data. This was followed by a *post hoc* multilevel pairwise comparison from package ‘vegan’ (Oksanen et al. 2020) in R Studio®. To determine the characteristic VOCs of each dung type, an IndVal analysis (Dufrêne and Legendre 1997) was applied to the GC/MS data for each dung type.

Both the behavioral and EAG data were checked for normality with Shapiro-Wilk Tests (α = 0.05). Differences in median value responses for both datasets were analyzed using Kruskal-Wallis rank sum tests (α = 0.05) given that both datasets exhibited heteroscedasticity in their standard deviations. Following significant differences in behavioral responses, *post hoc* Dunn tests (α = 0.05) for multiple pairwise comparisons were performed.

To determine the relationships between food preferences and the EAG profiles, we performed a Permutational Analysis of Variance (PERMANOVA) with the Bray-Curtis dissimilarity (BCD) matrix obtained from the EAG data using ‘Feeding preference’ as a factor. *Post-hoc* pairwise comparisons among groups were obtained by calculating a pseudo-F statistic for each treatment and *P* values estimated by using a permutation procedure (9999 iterations in this study) followed by a Bonferroni correction to the *P* values. For a graphical illustration of the differences detected we ran a Canonical Variates Analysis (CVA) (Lavine and Rayens 2009) applied to the physiological data grouped by factor ‘Feeding preference’. To determine which compounds were primarily responsible for the differences among feeding preference groups, a Similarity Percentages analysis (SIMPER) was also tested using the Bray-Curtis dissimilarity measure (Clarke 1993) and radar plots were made to display multivariate data in the form of a two-dimensional chart showing the antennal response of each feeding preference group to each EAG-active compound. These analyses were performed using PAST software (Hammer et al. 2001).

## Results

*Chemical Relationships Among Cow, Horse, and Rabbit Dung.* VOC profiles differed among dung types, both quantitatively and qualitatively (PERMANOVA on BCD, permutations = 9999, *df* = 2, *pseudo-F* = 15.00, *P* = 0.004). In total, we found 51 different compounds, 18 of which were found in rabbit, 27 in cow, and 40 in horse dung (Table2). We also identified 30 VOCs associated with a particular dung type (IndVal > 0.45, *P* < 0.05; see Table2). We found compounds with significant IndVal values exclusive to the different dung types, including 1H-indole for cow, skatole, acetophenone, and undecane for horse, and verbenone, 1,8-cineole, and camphene for rabbit dung. Other compounds were characteristic of both cow and horse dung, such as ρ-cresol and 6-methyl-5-hepten-2-one. Finally, some compounds with significant IndVal were shared among all three dung types, such as ρ-cymene, nonanal, β-caryophyllene, sabinene, and γ-terpinene.

**Table 2:** Chemical composition of different dung types analyzed by headspace sorptive extraction-gas chromatography/mass spectrometry

Compound^a^	Family group	RI^b^	RI^c^	Identified^d^	Composition (%)^e^		
					Dung		
					Cow	Horse	Rabbit
**2-Heptanone**	Ketone	866	889	MS, RI, STD	ND	1.2*	ND
Nonane	Hydrocarbon	889	900	MS, RI, STD	ND	0.3*	ND
Heptanal	Ketone	895	901	MS, RI, STD	0.5*	ND	ND
**α-Pinene**	Monoterpene	933	932	MS, RI, STD	8.6*	ND	8.2*
**Camphene**	Monoterpene	939	946	MS, RI, STD	ND	ND	4.1*
(*E*)-2-Heptenal	Aldehyde	946	947	MS, RI	1.4	1.0	ND
**Sabinene**	Monoterpene	970	969	MS, RI, STD	3.1*	4.6*	1.7*
**6-Methyl-5-hepten-2-one**	Ketone	981	981	MS, RI, STD	2.2*	4.7*	ND
2-Octanone	Ketone	986	988	MS, RI	ND	2.0*	ND
3-Octanol	Alcohol	993	988	MS, RI	ND	0.9*	ND
**p** ***-*** **Cymene**	Monoterpene	1019	1020	MS, RI, STD	7.3*	1.2*	5.8*
Limonene	Monoterpene	1023	1024	MS, RI, STD	2.2*	1.6*	2.2*
**1,8-Cineole (eucalyptol)**	Monoterpene	1029	1026	MS, RI, STD	ND	ND	9.4*
**γ-Terpinene**	Monoterpene	1056	1054	MS, RI, STD	2.0	2.5	1.8
**Acetophenone**	Ketone	1064	1059	MS, RI, STD	ND	6.9*	ND
**ρ-Cresol**	Phenol	1079	1071	MS, RI, STD	28.0*	25.0*	ND
**2-Nonanone**	Ketone	1096	1087	MS, RI, STD	ND	0.6*	ND
**Undecane**	Hydrocarbon	1100	1100	MS, RI, STD	ND	1.4*	ND
**Nonanal**	Aldehyde	1104	1100	MS, RI, STD	2.7*	1.1*	2.2*
**Camphor**	Monoterpene	1145	1141	MS, RI, STD	ND	ND	5.3*
Isopinocamphone	Monoterpene	1176	1176	MS, RI	ND	ND	3.1*
2-Decanone	Ketone	1193	1190	MS, RI	ND	0.3*	ND
Dodecane	Hydrocarbon	1197	1200	MS, RI, STD	ND	0.6*	ND
Decanal	Ketone	1205	1204	MS, RI, STD	0.9*	0.4*	ND
**Verbenone**	Monoterpene	1213	1204	MS, RI	ND	ND	14.3*
β-Cyclocitral	Monoterpene	1219	1217	MS, RI	1.6*	0.5*	0.7*
Benzothiazole	Miscellaneous	1229	Not RI	MS, RI, STD	ND	ND	4.5*
**1H-Indole**	Miscellaneous	1299	1290	MS, RI, STD	13.3*	ND	ND
2-Undecanone	Ketone	1300	1293	MS, RI	ND	0.7*	ND
α-Cubebene	Sesquiterpene	1355	1345	MS, RI, STD	0.1*	0.3*	3.5*
α-Ylangene	Sesquiterpene	1377	1373	MS, RI, STD	ND	1.2*	ND
α-Copaene	Sesquiterpene	1381	1374	MS, RI, STD	1.3*	1.8*	ND
**Skatole**	Miscellaneous	1391	1381	MS, RI, STD	ND	5.2*	ND
β-Bourbonene	Sesquiterpene	1386	1387	MS, RI, STD	0.9*	ND	ND
Longifolene	Sesquiterpene	1410	1407	MS, RI, STD	ND	ND	8.0*
**(** ***E*** **)-β-Caryophyllene**	Sesquiterpene	1420	1417	MS, RI, STD	10.9*	13.4*	8.9*
β-Copaene	Sesquiterpene	1429	1430	MS, RI, STD	ND	0.7*	ND
α-trans-Bergamotene	Sesquiterpene	1435	1432	MS, RI	ND	0.6*	ND
Dihydro-β-ionone	Ketone	1439	1434	MS, RI, STD	ND	0.3*	ND
α-Humulene	Sesquiterpene	1452	1452	MS, RI, STD	1.5*	1.9*	6.2*
9-epi-(E)-Caryophyllene	Miscellaneous	1465	1464	MS, RI	ND	0.4*	ND
cis-Muurola-4(14),5-diene	Sesquiterpene	1468	1465	MS, RI	4.7*	1.6*	ND
γ-Himachalene	Sesquiterpene	1474	1481	MS, RI, STD	ND	1.2*	ND
Germacrene D	Sesquiterpene	1484	1484	MS, RI, STD	ND	1.3*	ND
**(** ***E*** **)-β-ionone**	Sesquiterpene	1487	1487	MS, RI, STD	1.3*	1.1*	ND
Valencene	Sesquiterpene	1488	1496	MS, RI	1.4*	3.6*	ND
Pentadecane	Hydrocarbon	1494	1500	MS, RI, STD	1.2*	3.8*	ND
β-Bisabolene	Sesquiterpene	1504	1505	MS, RI	0.6*	1.4*	ND
Tridecanal	Ketone	1506	1509	MS, RI	0.8*	ND	ND
γ-Cadinene	Sesquiterpene	1511	1513	MS, RI, STD	0.6*	0.9*	ND
δ-Cadinene	Sesquiterpene	1527	1522	MS, RI, STD	0.3*	1.3*	10.2*
Tetradecanal	Ketone	1620	1611	MS, RI	0.6*	0.3*	ND
Total compounds					27	40	18

^a^Volatile organic compounds in order of elution on a polar DB5 capillary column

^b^Retention indices determined using the homologous series of n-alkanes (C7–C30)

^c^Retention indices obtained using data from the literature (Adams 2017)

^d^Method of identification: MS, identified by comparison with mass spectral databases; RI, identified by retention indices; STD, comparison with the retention times and mass spectra of available standards

^e^Relative abundance calculated from mass chromatogran peak areas

ND, not detected. *Statistically significant IndVal scores (IndVal of at least 0.45 and *P* < 0.05)

Volatile compounds in bold were those selected for EAG assays

*Feeding Preference Behavior.* The olfactometer tests showed five groups of species with different feeding preferences (see Table1). The first group, comprising *Ammoecius elevatus*, *Onthophagus maki*, and *Ateuchetus cicatricosus*, showed a preference to cow dung (*P* < 0.05, *P* < 0.001, and *P* < 0.001, respectively; for statistical details see Online Resource Fig. S1). Adults of *Copris hispanus*, *O. emarginatus*, and *Typhaeus typhoeus* preferred horse dung (*P* < 0.01, *P* < 0.01, and *P* < 0.001, respectively; for statistical details see Online Resource Fig. S1), two species, *Anomius baeticus* and *Thorectes valencianus*, preferred rabbit dung (*P* < 0.01, and *P* < 0.001, respectively; for statistical details see Online Resource Fig. S1), while, a group comprising *Aphodius fimetarius, Bubas bison*, *O. fracticornis*, *O. melitaeus*, *Ceratophyus hoffmannseggi* and *Sericotrupes niger* had equal preference to both cow and horse dung (*P* < 0.05, *P* < 0.01, *P* < 0.05, *P* < 0.001, *P* < 0.01, and *P* < 0.001, respectively; for statistical details see Online Resource Fig. S1). Finally, *Jekelius hernandezi* exhibited similar attraction to all three dung types (*P* = 0.35; see Online Resource Fig. S1).

**Fig. 1:**
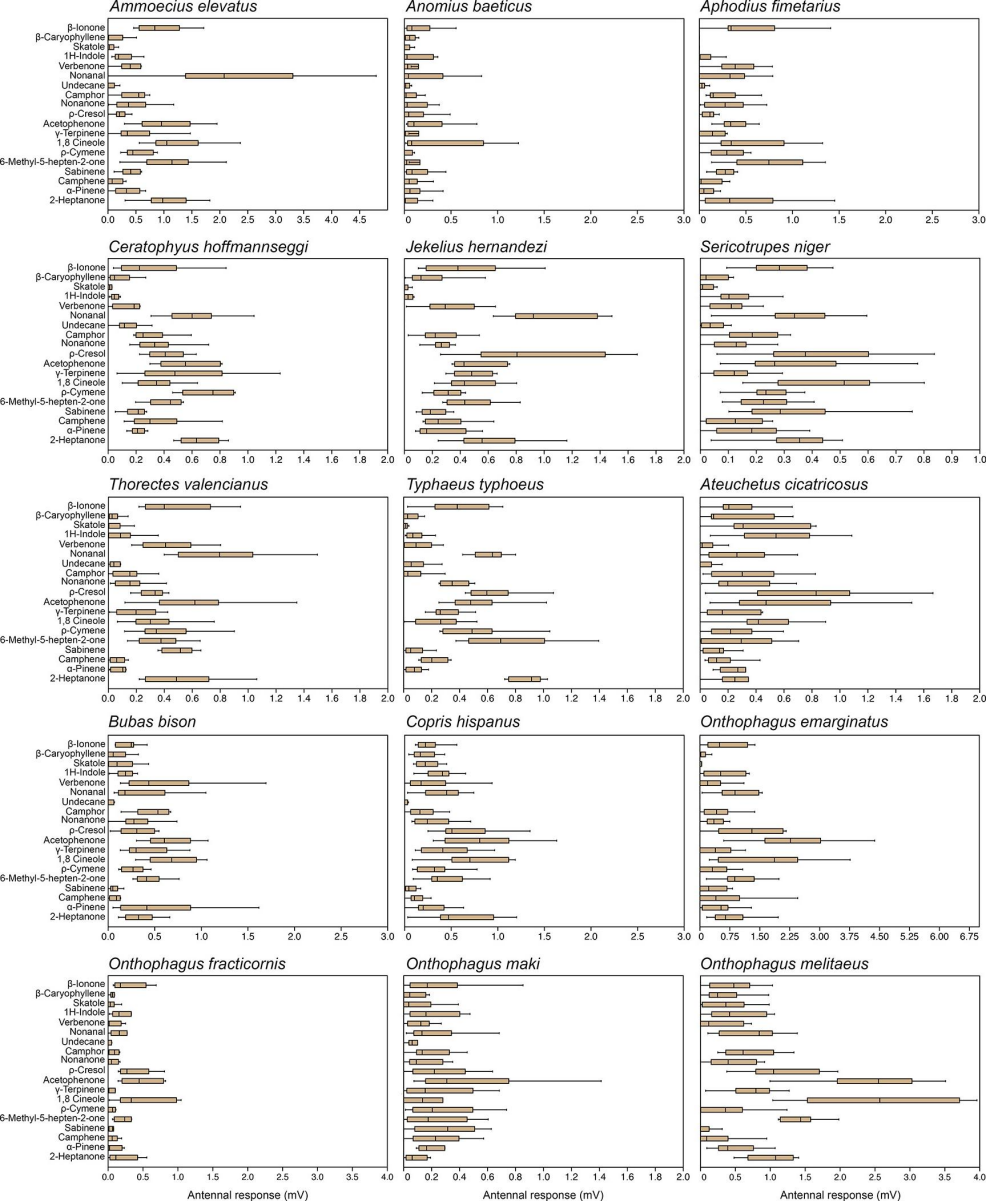
Eletroantennogram responses of dung beetles to volatile compounds of the different dung types analyzed. For statistical analysis see Online Resource Table S1

*Electroantennography Responses to VOCs.* Having established trophic preferences of the dung beetles to the different dung types, we studied the olfactory basis of this attraction. A group of 19 compounds selected from the VOCs emitted by the three dung types was tested individually. EAG responses revealed that dung beetles responded to all compounds, albeit with diverse profiles among species (Fig.1 and Online Resource Table S1).

**Fig. 2:**
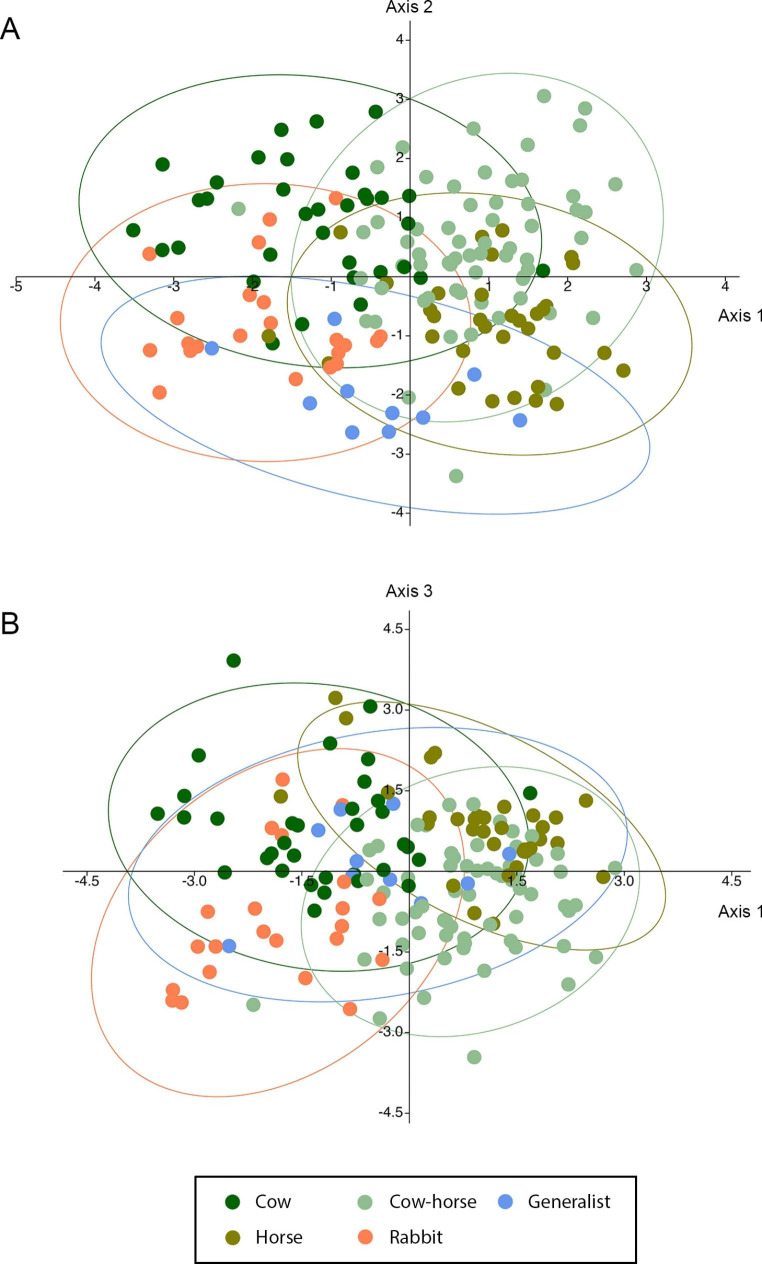
Plots of the Canonical Variates Analysis of eletroantennogram (EAG) responses of 15 dung beetle species grouped by ‘Feeding preference’ factor on the first three canonical variates, resulting from the application of stepwise discriminant function analysis to 19 EAG-active compounds. (A) Canonical variates 1 and 2 showing 42.9% of the total variation. (B) Canonical variates 1 and 3 showing 24.4% of the total variation

The relationships between behavioral preference and EAG profiles were plotted using a Canonical Variates Analysis of the EAG responses of 15 dung beetle species grouped by the ‘Feeding preference’ factor resulting from the application of stepwise discriminant function analysis to 19 EAG-active compounds (the three canonical variates represent 67.3% of the total variation; see Fig.2). PERMANOVA analysis revealed that dung beetles with different feeding preferences had differences in EAG responses to the compounds (PERMANOVA on BCD, permutations = 9999, *df* = 4, *pseudo-F* = 4.95, *P* < 0.001). EAG responses elicited by all five feeding-preference groups of dung beetles were different from each other in pairwise comparisons (Bonferroni-corrected *P* < 0.05, in all cases).

**Fig. 3:**
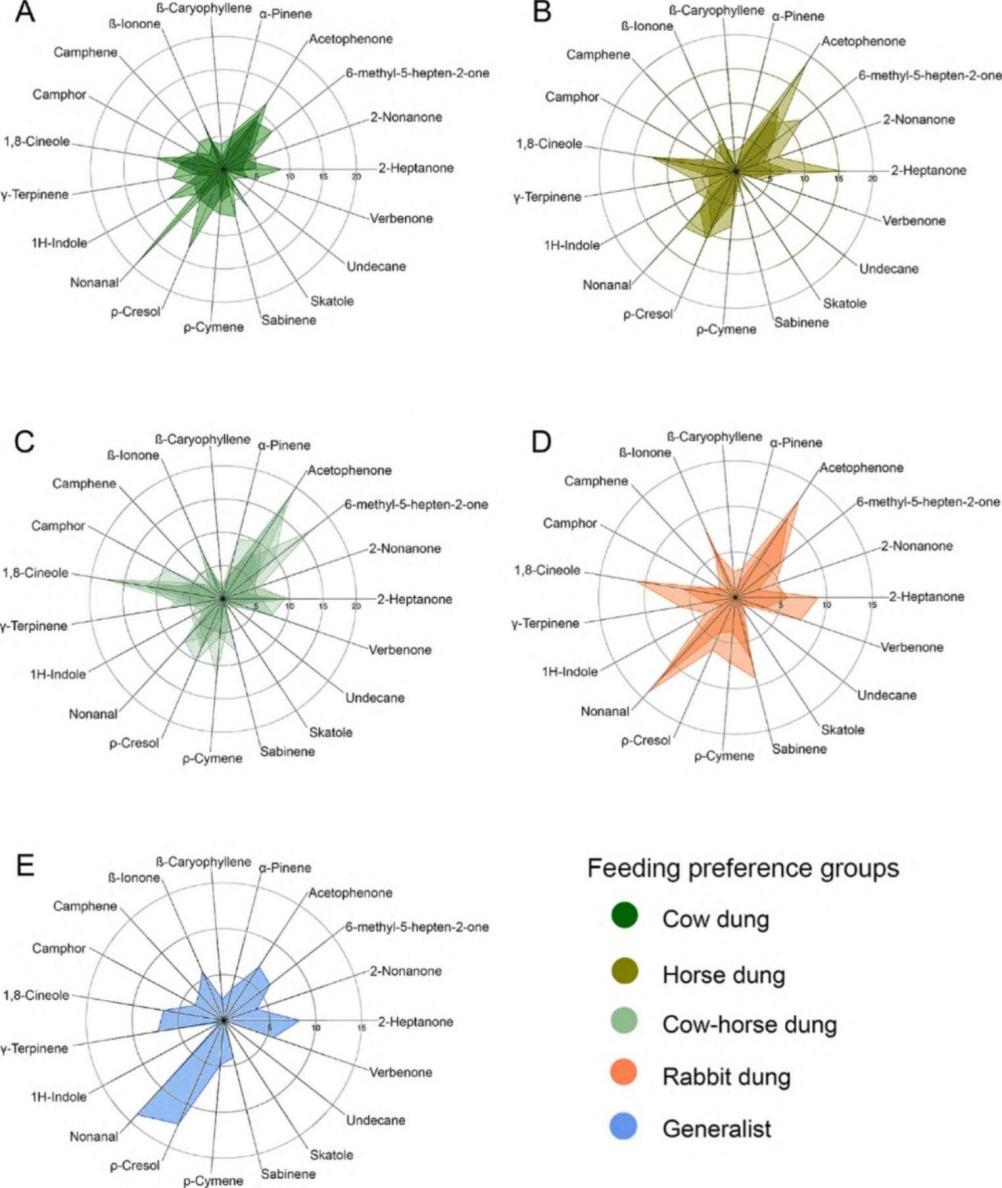
Radar charts comparing dung beetle eletroantennogram profiles of the different feeding preference groups. For dissimilarity and contribution percentages that each compound contributes among the five feeding preference groups see Table3

Considering the 19 EAG-active compounds, radar plots showed different sensitivity EAG profiles for each of the feeding groups (Fig.3). The SIMPER analysis, performed to explore if distinct sensory profiles of the groups of species can be attributed to a specific or set of EAG-active compounds, showed that a subset of compounds elicited strong dissimilarities among the feeding preference groups (Table3). Of these EAG responses, nonanal and sabinene were associated with species with a preference for rabbit dung, acetophenone, ρ-cresol, 2-heptanone, and 6-methyl-5-hepten-2-one to species with a preference to horse dung and 1H-indole to species with a preference for cow dung. EAG responses to 6-methyl-5-hepten-2-one were also relevant to species with a preference for both cow and horse dung. Finally, nonanal and ρ-cresol were associated with *J. hernandezi*, the only species considered a generalist.

**Table 3:** Calculated volatile compound contribution to dissimilarity among the feeding preference groups of dung beetles

VOCs^a^	Dissimilarity^b^	Contribution (%)^c^	Rabbit	Cow	Horse	C-H^d^	G^e^
Nonanal	2.55	10.22	13.10	9.98	8.22	6.74	14.00
1,8-Cineole	2.25	9.02	8.40	8.17	9.66	12.30	6.63
ρ-Cresol	2.13	8.54	5.20	7.00	10.20	6.51	12.30
Acetophenone	2.08	8.35	11.60	10.10	12.90	11.80	6.98
Sabinene	1.63	6.55	7.99	4.59	1.89	3.35	4.18
2-Heptanone	1.62	6.48	7.42	5.49	9.80	7.51	8.25
6-methyl-5-hepten-2-one	1.40	5.63	6.41	6.53	8.68	8.72	6.35
1H-Indole	1.37	5.50	2.69	5.52	4.05	2.81	0.41
Verbenone	1.30	5.21	5.55	3.14	2.79	4.05	5.69
ρ-Cymene	1.14	4.59	5.47	5.24	5.13	5.55	4.85
Camphor	1.07	4.31	2.14	4.60	2.39	5.38	3.50
ß-Ionone	1.06	4.23	7.00	5.84	4.84	5.85	5.85
γ-Terpinene	1.01	4.06	4.71	5.33	5.10	4.75	7.27
α-Pinene	0.95	3.80	2.54	4.56	2.86	4.31	3.51
Skatole	0.94	3.75	1.35	3.37	1.64	1.10	0.258
Camphene	0.83	3.32	1.86	3.17	3.18	2.63	3.98
2-Nonanone	0.61	2.43	3.63	4.09	4.69	4.26	3.75
ß-Caryophyllene	0.60	2.43	1.76	2.20	1.40	1.28	2.28
Undecane	0.39	1.58	1.23	1.04	0.58	1.12	0.01

^a^ The volatile organic compounds used in the electroantennogram bioassays in order of importance at contributing to the dissimilarity among the feeding preference groups

^b^ The average dissimilarity that each compound contributes among the five feeding preference groups

^c^ The percentage of contribution that each compound has on the separation of the preference groups

^d^ Cow-Horse feeding preference group

^e^ Generalist feeding preference group

## Discussion

### Volatile Chemical Profile of Dung Types and Feeding Preferences in Dung Beetles

Each dung type showed a diverse and characteristic assemblage of VOCs, including hydrocarbons, aldehydes, ketones, alcohols, phenols, monoterpenes, sesquiterpenes and a miscellaneous group of compounds that differed both quantitatively and qualitatively. Among these compounds, ρ-cresol, 1H-indole, and skatole are some of the most frequently cited dung volatiles in olfactory studies on dung beetles (Dormont et al. 2010; Frank et al. 2018b; Stavert et al. 2014). As found in previous studies, ρ-cresol was the most abundant compound in cow (28%) and horse (25%) dung (Table2). As such, the chemical profile of the VOCs identified in cow and horse dung are likely representative of freshly excreted dung samples, comprised primarily of anaerobically produced volatiles along with several mono- and sesquiterpenes that are normally obtained from a variety of shrub and pasture species (Elegbede and Gould 2002; Estell et al. 2008). Though not detected in this study, 1H-indole has previously been identified in horse dung (Hough et al. 2018), as well as various domesticated (DeMoss and Moser 1969; Martineau and Laflamme 2002) and non-domesticated mammals (Stahl et al. 2015; Vester et al. 2008). Though undecane was found only in horse dung, previous studies have identified this in various farm animals (Aii et al. 1980; Hobbs et al. 1996; Murphy et al. 2012), as well as in human, excrement (Raman et al. 2013). It is important to note that some of the VOCs identified in cow and horse dung were also found in rabbit dung, even though it had the lowest number of compounds (Table2, see also Goodrich et al. 1981). For example, nonanal was found in low abundance in all dung types examined. This compound has been found in several dung types, including dog, white rhino, red deer, wild boar, fox, and sheep (Arnould et al. 1998; Dormont et al. 2010; Frank et al. 2018b; Marneweck et al. 2018). The compound 6-methyl-5-hepten-2-one, absent in rabbit dung, has been identified in various other dungs (Marneweck et al. 2017; Murphy et al. 2012; Raman et al. 2013, see also Goodrich et al. 1981). Rabbit dung was the most chemically distinctive, with several exclusive VOCs, such as the monoterpenes camphene, 1,8-cineole, and verbenone (Table2) (see Couch et al. 2015; Marneweck et al. 2017; Pillai et al. 2010; Raman et al. 2013). Interestingly, rabbit dung contained a high content of 1,8-cineole, which is considered a toxin to some herbivorous marsupials, necessitating metabolism and excretion (McLean and Foley 1997). Furthermore, in some phytophagous beetles, such as the Christmas beetle, *Anoplognatus montanus* (Coleoptera: Rutelidae), the Mountain pine beetle, *Dendroctonus ponderosae* (Coleoptera: Curculionidae), and the Colorado potato beetle, *Leptinotarsa decemlineata* (Coleoptera: Chrysomelidae), the monoterpenes 1,8-cineole and verbenone have high antifeedant activity (Edwards et al. 1993; Ortiz de Elguea-Culebras et al. 2017).

The high heterogeneity observed among the chemical profiles of the different dung types suggests that food selection behavior by dung beetles may be determined by olfactory discrimination. Other studies on attraction of dung beetles to different dung types have provided evidence for feeding preferences (Dormont et al. 2004, 2007, 2010; Galante and Cartagena 1999; Lumaret and Iborra 1996; Martín-Piera and Lobo 1996). However, our olfactometer bioassays demonstrated that most species in our study do not show exclusive selection for one type of dung, which partially supports the hypothesis of ‘*choosy generalism*’ in coprophagous dung beetles (Dormont et al. 2004, 2007; Frank et al. 2018b). Despite the different chemical profiles among the three types of dung, a high number of dung beetle species showed similar preferences toward cow and horse dung. This last finding may be supported by the high degree of similarity between the chemical profiles of cow and horse dung (see Table2). Although most species presented a ‘*choosy generalism*’ pattern in attraction to the different feces, a few species showed a preference for a single food resource. Among them, the predilection of *Anomius baeticus* and *Thorectes valencianus* for rabbit dung supports the association of these species with rabbit latrines (Verdú and Galante 2004). This suggests that these species have a more specialized strategy, associated with their modified mouthparts, as rabbit dung is hard and dry (Verdú and Galante 2004).

### Feeding Preferences in Dung Beetles May be Mediated by Electrophysiological Responses to VOCs

EAG response profiles to the different chemicals differed among the dung beetle species, especially among species with different feeding preferences to three dung sources. This suggests that trophic preferences in dung beetles are not mediated by single VOCs, but rather through recognition of volatile mixtures. Although individual VOCs can trigger attraction towards a food resource, insects more commonly respond to complex mixtures (Clifford and Riffell 2013; Riffell et al. 2009; Riffell 2012). A field study on attraction of dung beetles using different food odor mixtures and single components revealed little to no attraction to most of the single components, with the more complex mixtures giving higher attraction (Frank et al. 2018b). Our EAG results again support the hypothesis of ‘*choosy generalism*’ in dung beetles. Having the ability to detect a broad range of dung VOCs, many of them characteristic of different types of dung, could allow dung beetles to locate a great variety of food resources. Given that excrements are usually ephemeral and stochastic resources (Hanski and Cambefort 1991), it may be that responding to a blend of compounds is more reliable than a single VOC specific to a given type of dung.

It is likely that VOCs from intestinal anaerobe metabolism of amino acids (Mackie et al. 1998) are common to many types of dung. Some of these compounds, such as ρ-cresol, the product of tyrosine fermentation, and 1H-indole and skatole, end products of tryptophan metabolism (Saito et al. 2018), may be candidates for a possible ‘*generalized dung bouquet*’. A generalized response to such compounds across species supports the results of the olfactometer tests, in which many species showed a similar preference for cow and horse dung. Our EAG results showed strong dung beetle responses to ρ-cresol, 1H-indole, and skatole, but also suggested that other compounds, such as nonanal, acetophenone, and ρ-cymene, may also be involved in a ‘*generalized dung bouquet*’. Acetophenone, which was only identified in horse dung in our study, has previously been identified in dung samples of cows (Laor et al. 2008), pigs (Blanes-Vidal et al. 2009), and several wild vertebrates (Apps et al. 2012; Marneweck et al. 2017; Martín et al. 2010; Stahl et al. 2015), indicating that this may also be a general component of dung. Other electrophysiological and behavioral studies on dung beetles have shown the importance of compounds such as ρ-cresol, indole, and skatole in dung beetle responses (Frank et al. 2018b; Inouchi et al. 1988; Shibuya and Inouchi, 1982; Weithmann et al. 2020).

Although certain compounds could be part of a ‘*generalized dung bouquet*’, acting as a common attractant to a large number of dung beetle species, our results also suggested that some compounds could determine preferences for a particular dung. For example, acetophenone, 2-heptanone, and 6-methyl-5-hepten-2-one may influence beetles to select horse dung (Table3). Additionally, nonanal, sabinene, and verbenone may function as key components to attract specialized species such as *Thorectes valencianus* and *Anomius baeticus* to rabbit dung (Table3).

## Conclusion

Chemical analyses showed that dung odor is composed of many components. Although the blend is often dominated by a few main components, this does not necessarily mean that these components provide the most important signal to dung beetles. Our combination of behavioral and physiological bioassays suggested the existence of key components for a possible ‘*generalized dung bouquet*’, as well as suggesting that some compounds could be involved in determining preferences for specific dung types. To further understand the role of key VOCs of dung it is crucial to conduct more detailed behavioral and electrophysiological studies to understand their biological, ecological, and evolutionary significance t**o** dung beetles.

## Electronic Supplementary Material

Below is the link to the electronic supplementary material.

Supplementary Material 1

Supplementary Material 2

## Data Availability

Data can be made available upon reasonable request.
