# Supplementary material for: Links Between Feeding Preferences and Electroantennogram Response Profiles in Dung Beetles: The Importance of Dung Odor Bouquets
Source: J Chem Ecol. 2022 Sep 9;48(9-10):690–703. doi: 10.1007/s10886-022-01383-1 (PMC9618527; doi:10.1007/s10886-022-01383-1)
Supplement: Supplementary file 1 — Supplementary Material 1 [file 10886_2022_1383_MOESM1_ESM.docx]

Journal of Chemical Ecology

Links between Feeding Preference and Electrophysiological Olfactory Profiles in Dung Beetles: The Importance of Dung Odor Bouquets

Miguel A. Urrutia, Vieyle Cortez, José R. Verdú

*Research Institute CIBIO (Centro Iberoamericano de la Biodiversidad) Science Park, University of Alicante, Alicante, E-03690, Spain.*


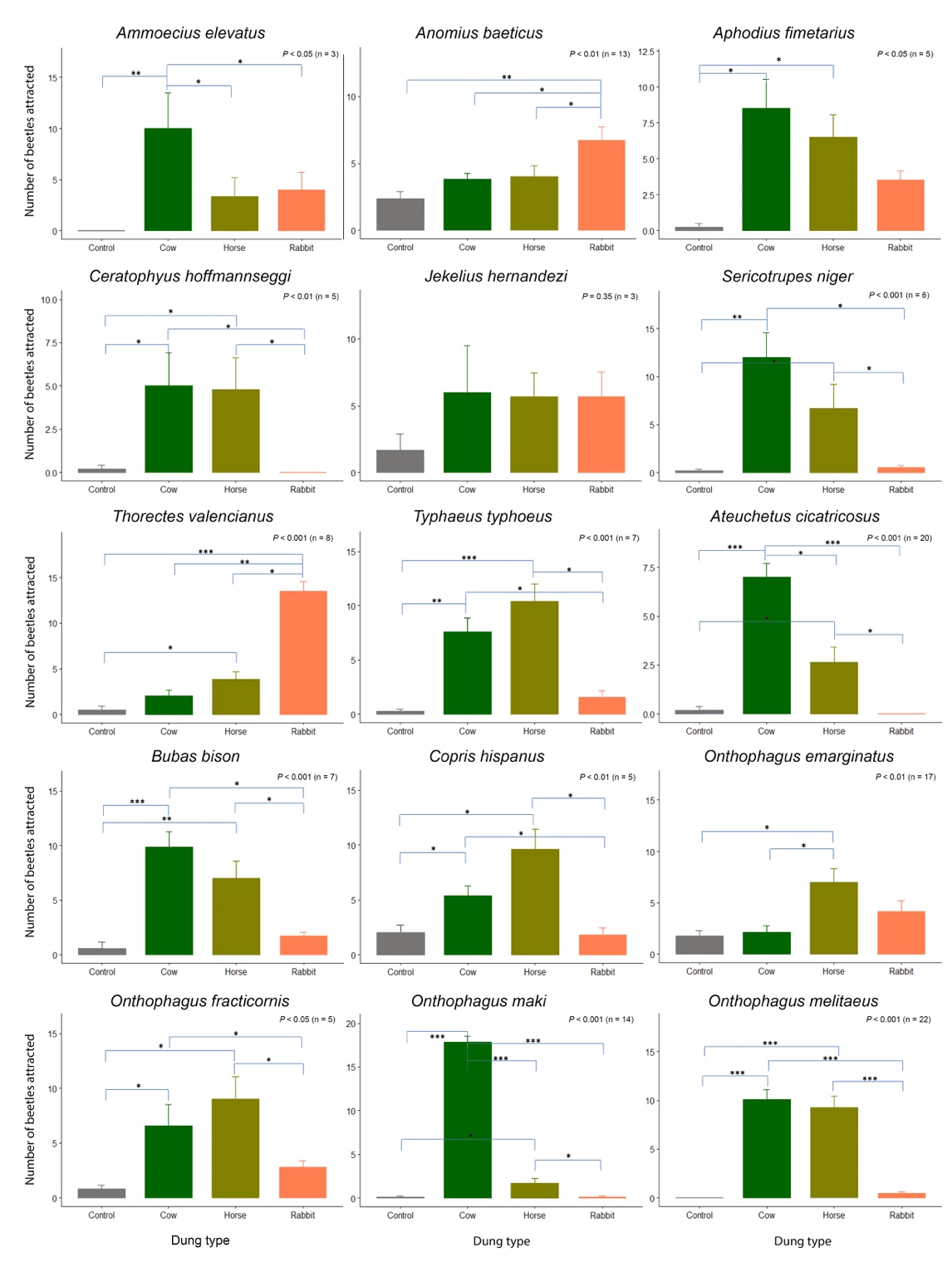


**Figure S1:** Food preference by dung beetles according to the olfactometry bioassays for each species of dung beetle. The Kruskal-Wallis test *P*-values denotes the global significance in the differences in attraction observed for each dung type and the control. The *n* denotes the number of replicates. The vertical lines on each bar represent the standard errors. The asterisks (*****) represent the significant differences between the trophic options according to the *post hoc* Dunn tests (* *P* < 0.05, ** *P* < 0.01, *** *P* < 0.001).
