# Supplementary material for: Links Between Feeding Preferences and Electroantennogram Response Profiles in Dung Beetles: The Importance of Dung Odor Bouquets
Source: J Chem Ecol. 2022 Sep 9;48(9-10):690–703. doi: 10.1007/s10886-022-01383-1 (PMC9618527; doi:10.1007/s10886-022-01383-1)
Supplement: Supplementary file 2 — Supplementary Material 2 [file 10886_2022_1383_MOESM2_ESM.docx]

Journal of Chemical Ecology

Links between Feeding Preference and Electrophysiological Olfactory Profiles in Dung Beetles: The Importance of Dung Odor Bouquets

Miguel A. Urrutia, Vieyle Cortez, José R. Verdú

*Research Institute CIBIO (Centro Iberoamericano de la Biodiversidad) Science Park, University of Alicante, Alicante, E-03690, Spain.*

**Table S1:** *Post hoc* multilevel pairwise comparisons of the electroantennography (EAG) bioassays.

*Ammoecius elevatus*, Kruskal –Wallis *P* < 0.001, Statistic: 142.908, N = 12

| **Volatile 1** | **Volatile 2** | **Statistic** | **P-value** | **P-value adjusted** | **Significance level** |
| --- | --- | --- | --- | --- | --- |
| 6-me-5-hepten-2-one | beta-Caryophyllene | -4.849316049 | 1.24E-06 | 0.000151143 | *** |
| 6-me-5-hepten-2-one | Camphene | -4.738553655 | 2.15E-06 | 0.000260451 | *** |
| 6-me-5-hepten-2-one | Indole | -3.499399376 | 0.000466 | 0.04989491 | * |
| 6-me-5-hepten-2-one | para-Cresol | -3.696694889 | 0.000218 | 0.02424513 | * |
| 6-me-5-hepten-2-one | Skatole | -5.148720644 | 2.62E-07 | 3.33E-05 | **** |
| 6-me-5-hepten-2-one | Undecane | -5.396205367 | 6.81E-08 | 8.85E-06 | **** |
| 2-heptanone | beta-Caryophyllene | -4.815617781 | 1.47E-06 | 0.000225988 | *** |
| 2-heptanone | Camphene | -4.725808832 | 2.29E-06 | 0.000348386 | *** |
| 2-heptanone | para-Cresol | -3.719329231 | 0.0002 | 0.028165109 | * |
| 2-heptanone | Skatole | -5.102077359 | 3.36E-07 | 5.34E-05 | **** |
| 2-heptanone | Undecane | -5.342084033 | 9.19E-08 | 1.51E-05 | **** |
| Acetophenone | beta-Caryophyllene | -4.683172458 | 2.82E-06 | 0.000338962 | *** |
| Acetophenone | Camphene | -4.572410064 | 4.82E-06 | 0.000568933 | *** |
| Acetophenone | para-Cresol | -3.530551299 | 0.000415 | 0.04520171 | * |
| Acetophenone | Skatole | -4.982577053 | 6.27E-07 | 7.78E-05 | **** |
| Acetophenone | Undecane | -5.230061777 | 1.69E-07 | 2.17E-05 | **** |
| Eucalyptol | Indole | -3.887067753 | 0.000101 | 0.011465256 | * |
| Eucalyptol | para-Cresol | -4.084363267 | 4.42E-05 | 0.005038554 | ** |
| Eucalyptol | Skatole | -5.536389022 | 3.09E-08 | 4.04E-06 | **** |
| Eucalyptol | Undecane | -5.783873745 | 7.30E-09 | 9.78E-07 | **** |
| beta-Ionone | Camphene | -4.46857032 | 7.87E-06 | 0.000913432 | *** |
| beta-Ionone | Skatole | -4.878737309 | 1.07E-06 | 0.000131324 | *** |
| beta-Ionone | Undecane | -5.126222033 | 2.96E-07 | 3.70E-05 | **** |
| Nonanal | para-Cresol | -4.501452906 | 6.75E-06 | 0.000789639 | *** |
| Nonanal | Skatole | -5.953478661 | 2.63E-09 | 3.54E-07 | **** |
| Nonanal | Undecane | -6.200963384 | 5.61E-10 | 7.63E-08 | **** |
| beta-Caryophyllene | beta-Ionone | 4.579332714 | 4.66E-06 | 0.000555089 | *** |
| beta-Caryophyllene | Eucalyptol | 5.236984426 | 1.63E-07 | 2.11E-05 | **** |
| beta-Caryophyllene | Nonanal | 5.654074065 | 1.57E-08 | 2.08E-06 | **** |
| Camphene | Eucalyptol | 5.126222033 | 2.96E-07 | 3.70E-05 | **** |
| Camphene | Nonanal | 5.543311671 | 2.97E-08 | 3.92E-06 | **** |
| para-Cymene | Skatole | -3.518436662 | 0.000434 | 0.046882522 | * |
| para-Cymene | Undecane | -3.765921385 | 0.000166 | 0.018584832 | * |
| Indole | Nonanal | 4.304157392 | 1.68E-05 | 0.001927658 | ** |
| alpha-Pinene | Nonanal | 3.68977224 | 0.000224 | 0.024690041 | * |

*Anomius baeticus*, Kruskal-Wallis, *P* = 0.191, Statistic: 22.985, N = 10

*Aphodius fimetarius*, Kruskal-Wallis, *P* < 0.001, Statistic: 103.602, N = 11

| **Volatile 1** | **Volatile 2** | **Statistic** | **P-value** | **P-value adjusted** | **Significance level** |
| --- | --- | --- | --- | --- | --- |
| 6-me-5-hepten-2-one | alpha-Pinene | -4.226227842 | 2.38E-05 | 0.002946753 | ** |
| 6-me-5-hepten-2-one | beta-Caryophyllene | -5.829965903 | 5.54E-09 | 7.54E-07 | **** |
| 6-me-5-hepten-2-one | Camphene | -3.897919182 | 9.70E-05 | 0.01183678 | * |
| 6-me-5-hepten-2-one | gamma-Terpinene | -3.675067243 | 0.000238 | 0.02805884 | * |
| 6-me-5-hepten-2-one | Indole | -4.512751763 | 6.40E-06 | 0.000825496 | *** |
| 6-me-5-hepten-2-one | para-Cresol | -3.732769977 | 0.000189 | 0.022726264 | * |
| 6-me-5-hepten-2-one | Skatole | -5.491708495 | 3.98E-08 | 5.37E-06 | **** |
| 6-me-5-hepten-2-one | Undecane | -4.663972722 | 3.10E-06 | 0.000403211 | *** |
| Acetophenone | beta-Caryophyllene | -4.791316687 | 1.66E-06 | 0.000220368 | *** |
| Acetophenone | Skatole | -4.45305928 | 8.47E-06 | 0.001075123 | ** |
| Acetophenone | Undecane | -3.625323507 | 0.000289 | 0.033477593 | * |
| beta-Ionone | Skatole | -4.429182286 | 9.46E-06 | 0.001191847 | ** |
| beta-Ionone | Undecane | -3.601446513 | 0.000316 | 0.036075475 | * |
| Eucalyptol | Skatole | -4.476936273 | 7.57E-06 | 0.00096924 | *** |
| Eucalyptol | Undecane | -3.6492005 | 0.000263 | 0.030777755 | * |
| 2-heptanone | beta-Caryophyllene | -4.106991194 | 4.01E-05 | 0.006333375 | ** |
| 2-heptanone | Skatole | -3.769478942 | 0.000164 | 0.025029073 | * |
| 2-Nonanone | beta-Caryophyllene | -3.877838138 | 0.000105 | 0.016335263 | * |
| beta-Caryophyllene | beta-Ionone | 4.767439694 | 1.87E-06 | 0.000246288 | *** |
| beta-Caryophyllene | Camphor | 3.609405511 | 0.000307 | 0.035293447 | * |
| beta-Caryophyllene | Eucalyptol | 4.815193681 | 1.47E-06 | 0.000197057 | *** |
| beta-Caryophyllene | Nonanal | 3.581549019 | 0.000342 | 0.038596623 | * |
| beta-Caryophyllene | para-Cymene | 3.808380456 | 0.00014 | 0.016925473 | * |
| beta-Caryophyllene | Sabinene | 4.059088888 | 4.93E-05 | 0.006059541 | ** |
| beta-Caryophyllene | Verbenone | 4.731624203 | 2.23E-06 | 0.000291777 | *** |
| Sabinene | Skatole | -3.72083148 | 0.000199 | 0.023629582 | * |
| Skatole | Verbenone | 4.393366796 | 1.12E-05 | 0.001395107 | ** |
| Undecane | Verbenone | 3.565631023 | 0.000363 | 0.040653957 | * |

*Bubas bison*, Kruskal-Wallis, *P* < 0.001, Statistic: 110.087, N=12

| **Volatile 1** | **Volatile 2** | **Statistic** | **P-value** | **P-value adjusted** | **Significance level** |
| --- | --- | --- | --- | --- | --- |
| Acetophenone | beta-Caryophyllene | -4.847196532 | 1.25E-06 | 0.000164036 | *** |
| Acetophenone | Camphene | -4.520360947 | 6.17E-06 | 0.000790199 | *** |
| Acetophenone | Indole | -3.721429517 | 0.000198 | 0.022781289 | * |
| Acetophenone | Sabinene | -5.149822073 | 2.61E-07 | 3.47E-05 | **** |
| Acetophenone | Skatole | -4.345702777 | 1.39E-05 | 0.001721498 | ** |
| Acetophenone | Undecane | -5.340043842 | 9.29E-08 | 1.25E-05 | **** |
| 6-me-5-hepten-2-one | beta-Caryophyllene | -3.927214885 | 8.59E-05 | 0.010226289 | * |
| 6-me-5-hepten-2-one | Camphene | -3.6003793 | 0.000318 | 0.034952864 | * |
| 6-me-5-hepten-2-one | Sabinene | -4.229840427 | 2.34E-05 | 0.002876443 | ** |
| 6-me-5-hepten-2-one | Undecane | -4.420062196 | 9.87E-06 | 0.001233406 | ** |
| Eucalyptol | Indole | -3.845938311 | 0.00012 | 0.014050761 | * |
| Eucalyptol | Sabinene | -5.274330867 | 1.33E-07 | 1.79E-05 | **** |
| Eucalyptol | Skatole | -4.470211571 | 7.81E-06 | 0.000992407 | *** |
| Eucalyptol | Undecane | -5.464552636 | 4.64E-08 | 6.31E-06 | **** |
| Camphor | Sabinene | -4.461565127 | 8.14E-06 | 0.001025176 | ** |
| Camphor | Skatole | -3.657445831 | 0.000255 | 0.028724037 | * |
| Camphor | Undecane | -4.651786896 | 3.29E-06 | 0.000427792 | *** |
| alpha-Pinene | Sabinene | -3.655716542 | 0.000256 | 0.028724037 | * |
| alpha-Pinene | Undecane | -3.845938311 | 0.00012 | 0.014050761 | * |
| beta-Caryophyllene | Camphor | 4.158939586 | 3.20E-05 | 0.003868714 | ** |
| beta-Caryophyllene | Eucalyptol | 4.971705326 | 6.64E-07 | 8.76E-05 | **** |
| beta-Caryophyllene | Verbenone | 3.716241651 | 0.000202 | 0.023051734 | * |
| Camphene | Camphor | 3.832104001 | 0.000127 | 0.014738033 | * |
| Camphene | Eucalyptol | 4.644869741 | 3.40E-06 | 0.000438976 | *** |
| gamma-Terpinene | Undecane | -3.638423654 | 0.000274 | 0.030448623 | * |
| Sabinene | Verbenone | 4.018867192 | 5.85E-05 | 0.007017434 | ** |
| Undecane | Verbenone | 4.209088961 | 2.56E-05 | 0.00312811 | ** |

*Copris hispanus*, Kruskal-Wallis, *P* < 0.001, Statistic: 112.057, N = 12

| **Volatile 1** | **Volatile 2** | **Statistic** | **P-value** | **P-value adjusted** | **Significance level** |
| --- | --- | --- | --- | --- | --- |
| Acetophenone | beta-Caryophyllene | -3.798945324 | 0.000145 | 0.017437577 | * |
| Acetophenone | Camphene | -4.896956376 | 9.73E-07 | 0.000126532 | *** |
| Acetophenone | Camphor | -4.084255283 | 4.42E-05 | 0.005438863 | ** |
| Acetophenone | Sabinene | -5.547117251 | 2.90E-08 | 3.86E-06 | **** |
| Acetophenone | Undecane | -6.28546484 | 3.27E-10 | 4.45E-08 | **** |
| Acetophenone | Verbenone | -3.629488501 | 0.000284 | 0.032090122 | * |
| 2-heptanone | Camphene | -3.935195701 | 8.31E-05 | 0.012801855 | * |
| 2-heptanone | Sabinene | -4.58203479 | 4.60E-06 | 0.000745967 | *** |
| 2-heptanone | Undecane | -5.276845198 | 1.31E-07 | 2.18E-05 | **** |
| 6-me-5-hepten-2-one | Sabinene | -3.826611745 | 0.00013 | 0.015720222 | * |
| 6-me-5-hepten-2-one | Undecane | -4.564959334 | 5.00E-06 | 0.000639477 | *** |
| Eucalyptol | Sabinene | -5.407055999 | 6.41E-08 | 8.46E-06 | **** |
| Eucalyptol | Undecane | -6.145403588 | 7.98E-10 | 1.08E-07 | **** |
| gamma-Terpinene | Sabinene | -3.747070787 | 0.000179 | 0.021290482 | * |
| gamma-Terpinene | Undecane | -4.485418376 | 7.28E-06 | 0.000924192 | *** |
| Indole | Sabinene | -3.736695879 | 0.000186 | 0.021815143 | * |
| Indole | Undecane | -4.475043468 | 7.64E-06 | 0.000954947 | *** |
| Nonanal | Sabinene | -3.743612484 | 0.000181 | 0.021404414 | * |
| Nonanal | Undecane | -4.481960073 | 7.40E-06 | 0.000931903 | *** |
| para-Cresol | Sabinene | -5.102725377 | 3.35E-07 | 4.39E-05 | **** |
| para-Cresol | Undecane | -5.841072966 | 5.19E-09 | 6.95E-07 | **** |
| beta-Caryophyllene | Eucalyptol | 3.658884072 | 0.000253 | 0.029131327 | * |
| Camphene | Eucalyptol | 4.756895124 | 1.97E-06 | 0.000253605 | *** |
| Camphene | para-Cresol | 4.452564502 | 8.49E-06 | 0.001052149 | ** |
| Camphor | Eucalyptol | 3.944194031 | 8.01E-05 | 0.009768395 | ** |
| Camphor | para-Cresol | 3.639863408 | 0.000273 | 0.031097228 | * |
| para-Cymene | Undecane | -3.674446434 | 0.000238 | 0.027650415 | * |

*Ceratophyus hoffmannseggi*, Kruskal-Wallis, *P* < 0.001, Statistic: 117.366, N = 10

| **Volatile 1** | **Volatile 2** | **Statistic** | **P-value** | **P-value adjusted** | **Significance level** |
| --- | --- | --- | --- | --- | --- |
| 2-heptanone | beta-Caryophyllene | -5.137756701 | 2.78E-07 | 4.59E-05 | **** |
| 2-heptanone | Indole | -5.03406632 | 4.80E-07 | 7.88E-05 | **** |
| 2-heptanone | Skatole | -5.871721944 | 4.31E-09 | 7.33E-07 | **** |
| 2-heptanone | Undecane | -4.53391272 | 5.79E-06 | 0.000920625 | *** |
| 2-heptanone | Verbenone | -4.088654027 | 4.34E-05 | 0.006595028 | ** |
| Acetophenone | beta-Caryophyllene | -4.531985971 | 5.84E-06 | 0.000742083 | *** |
| Acetophenone | Indole | -4.434304068 | 9.24E-06 | 0.00114539 | ** |
| Acetophenone | Skatole | -5.324799557 | 1.01E-07 | 1.35E-05 | **** |
| Acetophenone | Undecane | -3.875472715 | 0.000106 | 0.012557313 | * |
| para-Cymene | Sabinene | -3.53472189 | 0.000408 | 0.0449025 | * |
| para-Cymene | Skatole | -6.106254783 | 1.02E-09 | 1.39E-07 | **** |
| para-Cymene | Undecane | -4.656927941 | 3.21E-06 | 0.000410832 | *** |
| para-Cymene | Verbenone | -4.213951868 | 2.51E-05 | 0.003086572 | ** |
| 6-me-5-hepten-2-one | beta-Caryophyllene | -3.732357369 | 0.00019 | 0.022194445 | * |
| 6-me-5-hepten-2-one | Indole | -3.634675466 | 0.000278 | 0.031451378 | * |
| 6-me-5-hepten-2-one | Skatole | -4.525170955 | 6.03E-06 | 0.000760368 | *** |
| Nonanal | Skatole | -5.57922684 | 2.42E-08 | 3.26E-06 | **** |
| Nonanal | Undecane | -4.129899998 | 3.63E-05 | 0.004427637 | ** |
| Nonanal | Verbenone | -3.686923926 | 0.000227 | 0.026329816 | * |
| gamma-Terpinene | Indole | -3.889102748 | 0.000101 | 0.011973246 | * |
| gamma-Terpinene | Skatole | -4.779598238 | 1.76E-06 | 0.00022834 | *** |
| para-Cresol | Skatole | -4.434304068 | 9.24E-06 | 0.00114539 | ** |
| 2-Nonanone | Skatole | -3.614931308 | 0.0003 | 0.043261527 | * |
| beta-Caryophyllene | gamma-Terpinene | 3.986784652 | 6.70E-05 | 0.008103946 | ** |
| beta-Caryophyllene | Nonanal | 4.786413254 | 1.70E-06 | 0.000222423 | *** |
| beta-Caryophyllene | para-Cresol | 3.641490482 | 0.000271 | 0.030901313 | * |
| beta-Caryophyllene | para-Cymene | 5.313441197 | 1.08E-07 | 1.43E-05 | **** |
| Camphene | Skatole | -3.643762154 | 0.000269 | 0.030898398 | * |
| Camphor | Skatole | -3.550623596 | 0.000384 | 0.043043799 | * |
| Eucalyptol | Skatole | -3.975426291 | 7.03E-05 | 0.008430396 | ** |
| Indole | Nonanal | 4.688731351 | 2.75E-06 | 0.000354626 | *** |
| Indole | para-Cresol | 3.543808579 | 0.000394 | 0.043777454 | * |
| Indole | para-Cymene | 5.215759293 | 1.83E-07 | 2.42E-05 | **** |

*Jekelius hernandezi*, Kruskal-Wallis, *P* < 0.001, Statistic: 125.506, N= 10

| **Volatile 1** | **Volatile 2** | **Statistic** | **P-value** | **P-value adjusted** | **Significance level** |
| --- | --- | --- | --- | --- | --- |
| 2-heptanone | Indole | -4.831760112 | 1.35E-06 | 0.000217883 | *** |
| 2-heptanone | Skatole | -5.059519847 | 4.20E-07 | 6.85E-05 | **** |
| 2-heptanone | Undecane | -5.356420932 | 8.49E-08 | 1.40E-05 | **** |
| 6-me-5-hepten-2-one | Indole | -4.053844235 | 5.04E-05 | 0.005894784 | ** |
| 6-me-5-hepten-2-one | Skatole | -4.308345667 | 1.64E-05 | 0.001973762 | ** |
| 6-me-5-hepten-2-one | Undecane | -4.640106462 | 3.48E-06 | 0.000438769 | *** |
| Acetophenone | Indole | -4.35833702 | 1.31E-05 | 0.001585759 | ** |
| Acetophenone | Skatole | -4.612838452 | 3.97E-06 | 0.000496509 | *** |
| Acetophenone | Undecane | -4.944599247 | 7.63E-07 | 9.84E-05 | **** |
| beta-Ionone | Indole | -3.558475377 | 0.000373 | 0.040658499 | * |
| beta-Ionone | Skatole | -3.812976809 | 0.000137 | 0.015789845 | * |
| beta-Ionone | Undecane | -4.144737604 | 3.40E-05 | 0.004014396 | ** |
| Eucalyptol | Indole | -4.149282272 | 3.34E-05 | 0.00396888 | ** |
| Eucalyptol | Skatole | -4.403783704 | 1.06E-05 | 0.001297824 | ** |
| Eucalyptol | Undecane | -4.735544499 | 2.18E-06 | 0.000277454 | *** |
| gamma-Terpinene | Indole | -4.485587736 | 7.27E-06 | 0.000894373 | *** |
| gamma-Terpinene | Skatole | -4.740089167 | 2.14E-06 | 0.000273439 | *** |
| gamma-Terpinene | Undecane | -5.071849962 | 3.94E-07 | 5.12E-05 | **** |
| Nonanal | Sabinene | -3.792525801 | 0.000149 | 0.016999994 | * |
| Nonanal | Skatole | -6.285276432 | 3.27E-10 | 4.42E-08 | **** |
| Nonanal | Undecane | -6.617037227 | 3.66E-11 | 4.98E-09 | **** |
| para-Cresol | Skatole | -5.549040147 | 2.87E-08 | 3.79E-06 | **** |
| para-Cresol | Undecane | -5.880800942 | 4.08E-09 | 5.43E-07 | **** |
| 2-Nonanone | Nonanal | 3.656357189 | 0.000256 | 0.03658295 | * |
| alpha-Pinene | Nonanal | 3.697087764 | 0.000218 | 0.024643827 | * |
| beta-Caryophyllene | Nonanal | 4.608293783 | 4.06E-06 | 0.000503424 | *** |
| beta-Caryophyllene | para-Cresol | 3.872057498 | 0.000108 | 0.012518776 | * |
| Camphor | Nonanal | 3.644824077 | 0.000268 | 0.029968374 | * |
| Indole | Nonanal | 6.030775 | 1.63E-09 | 2.19E-07 | **** |
| Indole | para-Cresol | 5.294538715 | 1.19E-07 | 1.56E-05 | **** |
| para-Cymene | Undecane | -3.590288056 | 0.00033 | 0.036334399 | * |
| Undecane | Verbenone | 3.590288056 | 0.00033 | 0.036334399 | * |

*Onthophagus emarginatus*, Kruskal-Wallis, *P* < 0.001, Statistic: 93.250, N = 11

| **Volatile 1** | **Volatile 2** | **Statistic** | **P-value** | **P-value adjusted** | **Significance level** |
| --- | --- | --- | --- | --- | --- |
| Acetophenone | beta-Caryophyllene | -5.324188431 | 1.01E-07 | 1.36E-05 | **** |
| Acetophenone | para-Cymene | -3.858652165 | 0.000114 | 0.014023731 | * |
| Acetophenone | Sabinene | -3.979296851 | 6.91E-05 | 0.008639924 | ** |
| Acetophenone | Skatole | -5.626789037 | 1.84E-08 | 2.48E-06 | **** |
| Acetophenone | Undecane | -6.044100983 | 1.50E-09 | 2.04E-07 | **** |
| Acetophenone | Verbenone | -3.757785297 | 0.000171 | 0.020742288 | * |
| 6-me-5-hepten-2-one | beta-Caryophyllene | -3.753829733 | 0.000174 | 0.020898382 | * |
| 6-me-5-hepten-2-one | Skatole | -4.056430339 | 4.98E-05 | 0.00627838 | ** |
| 6-me-5-hepten-2-one | Undecane | -4.473742285 | 7.69E-06 | 0.000999211 | *** |
| Eucalyptol | Skatole | -4.705142748 | 2.54E-06 | 0.000334869 | *** |
| Eucalyptol | Undecane | -5.122454694 | 3.02E-07 | 4.01E-05 | **** |
| Nonanal | Skatole | -3.971385724 | 7.15E-05 | 0.008860516 | ** |
| Nonanal | Undecane | -4.38869767 | 1.14E-05 | 0.001459603 | ** |
| para-Cresol | Skatole | -4.143452735 | 3.42E-05 | 0.004344868 | ** |
| para-Cresol | Undecane | -4.560764681 | 5.10E-06 | 0.000667677 | *** |
| 2-heptanone | Undecane | -3.948094612 | 7.88E-05 | 0.012289005 | * |
| beta-Caryophyllene | Eucalyptol | 4.402542142 | 1.07E-05 | 0.001380168 | ** |
| beta-Caryophyllene | Nonanal | 3.668785119 | 0.000244 | 0.029000991 | * |
| beta-Caryophyllene | para-Cresol | 3.84085213 | 0.000123 | 0.014958171 | * |

*Onthophagus fracticornis*, Kruskal-Wallis, *P* < 0.001, Statistic: 55.443, N = 8

| **Volatile 1** | **Volatile 2** | **Statistic** | **P-value** | **P-value adjusted** | **Significance level** |
| --- | --- | --- | --- | --- | --- |
| Acetophenone | Undecane | -4.066511192 | 4.77E-05 | 0.006490218 | ** |
| Eucalyptol | Undecane | -3.658265363 | 0.000254 | 0.03402636 | * |
| para-Cresol | Undecane | -3.671023045 | 0.000242 | 0.03261351 | * |

*Onthophagus maki*, Kruskal-Wallis, *P* = 0.05, Statistic: 29.319, N = 11

*Onthophagus melitaeus*, Kruskal-Wallis, *P* < 0.001, 132.60, N = 11

| **Volatile 1** | **Volatile 2** | **Statistic** | **P-value** | **P-value adjusted** | **Significance level** |
| --- | --- | --- | --- | --- | --- |
| Acetophenone | alpha-Pinene | -3.627851859 | 0.000286 | 0.030579439 | * |
| Acetophenone | beta-Caryophyllene | -4.469608336 | 7.84E-06 | 0.000956028 | *** |
| Acetophenone | beta-Ionone | -3.813591551 | 0.000137 | 0.015339717 | * |
| Acetophenone | Camphene | -5.263941913 | 1.41E-07 | 1.83E-05 | **** |
| Acetophenone | Indole | -3.706890026 | 0.00021 | 0.0230802 | * |
| Acetophenone | para-Cymene | -4.165311393 | 3.11E-05 | 0.003668949 | ** |
| Acetophenone | Sabinene | -5.967381598 | 2.41E-09 | 3.21E-07 | **** |
| Acetophenone | Skatole | -4.469608336 | 7.84E-06 | 0.000956028 | *** |
| Acetophenone | Undecane | -6.556215941 | 5.52E-11 | 7.45E-09 | **** |
| Acetophenone | Verbenone | -4.991260237 | 6.00E-07 | 7.62E-05 | **** |
| 6-me-5-hepten-2-one | beta-Caryophyllene | -3.584380867 | 0.000338 | 0.035477268 | * |
| 6-me-5-hepten-2-one | Camphene | -4.378714444 | 1.19E-05 | 0.001444515 | ** |
| 6-me-5-hepten-2-one | Sabinene | -5.082154129 | 3.73E-07 | 4.81E-05 | **** |
| 6-me-5-hepten-2-one | Skatole | -3.584380867 | 0.000338 | 0.035477268 | * |
| 6-me-5-hepten-2-one | Undecane | -5.670988472 | 1.42E-08 | 1.87E-06 | **** |
| 6-me-5-hepten-2-one | Verbenone | -4.106032768 | 4.03E-05 | 0.004709395 | ** |
| Eucalyptol | Indole | -3.734553384 | 0.000188 | 0.020873395 | * |
| Eucalyptol | para-Cymene | -4.192974752 | 2.75E-05 | 0.003276309 | ** |
| Eucalyptol | Sabinene | -5.995044956 | 2.03E-09 | 2.73E-07 | **** |
| Eucalyptol | Skatole | -4.497271694 | 6.88E-06 | 0.000853504 | *** |
| Eucalyptol | Undecane | -6.583879299 | 4.58E-11 | 6.23E-09 | **** |
| Eucalyptol | Verbenone | -5.018923595 | 5.20E-07 | 6.65E-05 | **** |
| 2-heptanone | Camphene | -3.659808404 | 0.000252 | 0.034074527 | * |
| 2-heptanone | Sabinene | -4.368443445 | 1.25E-05 | 0.001914569 | ** |
| 2-heptanone | Undecane | -4.926869063 | 8.36E-07 | 0.000133692 | *** |
| para-Cresol | Sabinene | -4.33919536 | 1.43E-05 | 0.001716064 | ** |
| para-Cresol | Undecane | -4.928029704 | 8.31E-07 | 0.000104659 | *** |
| 2-Nonanone | Acetophenone | 3.984967625 | 6.75E-05 | 0.00998842 | ** |
| 2-Nonanone | Eucalyptol | 4.006173661 | 6.17E-05 | 0.009194833 | ** |
| alpha-Pinene | Eucalyptol | 3.655515217 | 0.000257 | 0.027976592 | * |
| beta-Caryophyllene | Eucalyptol | 4.497271694 | 6.88E-06 | 0.000853504 | *** |
| beta-Ionone | Eucalyptol | 3.841254909 | 0.000122 | 0.014056404 | * |
| Camphene | Eucalyptol | 5.291605271 | 1.21E-07 | 1.59E-05 | **** |
| Camphene | para-Cresol | 3.635755675 | 0.000277 | 0.029934029 | * |
| Camphor | Undecane | -3.837303001 | 0.000124 | 0.014056404 | * |
| gamma-Terpinene | Undecane | -3.868918268 | 0.000109 | 0.012681036 | * |
| Nonanal | Undecane | -3.837303001 | 0.000124 | 0.014056404 | * |

*Ateuchetus cicatricosus* Krskal-Wallis, *P* < 0.001, Statistic: 74.391, N = 11

| **Volatile 1** | **Volatile 2** | **Statistic** | **P-value** | **P-value adjusted** | **Significance level** |
| --- | --- | --- | --- | --- | --- |
| para-Cresol | Sabinene | -3.838539131 | 0.000124 | 0.015718599 | * |
| para-Cresol | Undecane | -5.604109491 | 2.09E-08 | 2.85E-06 | **** |
| para-Cresol | Verbenone | -5.056309758 | 4.27E-07 | 5.77E-05 | **** |
| Acetophenone | Undecane | -4.802114919 | 1.57E-06 | 0.000208808 | *** |
| Acetophenone | Verbenone | -4.254315186 | 2.10E-05 | 0.002725967 | ** |
| Eucalyptol | Undecane | -4.634622195 | 3.58E-06 | 0.000472019 | *** |
| Eucalyptol | Verbenone | -4.086822463 | 4.37E-05 | 0.005641447 | ** |
| Indole | Undecane | -4.973548648 | 6.57E-07 | 8.81E-05 | **** |
| Indole | Verbenone | -4.425748916 | 9.61E-06 | 0.001259018 | ** |
| Skatole | Undecane | -4.035589394 | 5.45E-05 | 0.006971571 | ** |

*Sericotrupes niger*, Kruskal-Wallis, *P* < 0.001, Statistic: 128.296, N = 12

| **Volatile 1** | **Volatile 2** | **Statistic** | **P-value** | **P-value adjusted** | **Significance level** |
| --- | --- | --- | --- | --- | --- |
| Eucalyptol | gamma-Terpinene | -4.069951979 | 4.70E-05 | 0.005454648 | ** |
| Eucalyptol | Indole | -3.99384578 | 6.50E-05 | 0.007476168 | ** |
| Eucalyptol | Skatole | -6.031416299 | 1.63E-09 | 2.21E-07 | **** |
| Eucalyptol | Undecane | -5.543644749 | 2.96E-08 | 4.00E-06 | **** |
| Eucalyptol | Verbenone | -4.384754895 | 1.16E-05 | 0.001439844 | ** |
| 2-heptanone | beta-Caryophyllene | -4.749195826 | 2.04E-06 | 0.000322679 | *** |
| 2-heptanone | Skatole | -5.270864337 | 1.36E-07 | 2.27E-05 | **** |
| 2-heptanone | Undecane | -4.81575887 | 1.47E-06 | 0.000236093 | *** |
| 2-heptanone | Verbenone | -3.664063403 | 0.000248 | 0.035250831 | * |
| Acetophenone | beta-Caryophyllene | -4.3207565 | 1.55E-05 | 0.001865942 | ** |
| Acetophenone | Skatole | -4.8656077 | 1.14E-06 | 0.000147196 | *** |
| Acetophenone | Undecane | -4.377836149 | 1.20E-05 | 0.00147432 | ** |
| Nonanal | Skatole | -5.246138697 | 1.55E-07 | 2.05E-05 | **** |
| Nonanal | Undecane | -4.758367146 | 1.95E-06 | 0.000245908 | *** |
| Nonanal | Verbenone | -3.599477292 | 0.000319 | 0.035074322 | * |
| para-Cresol | Skatole | -5.48137604 | 4.22E-08 | 5.61E-06 | **** |
| para-Cresol | Undecane | -4.99360449 | 5.93E-07 | 7.76E-05 | **** |
| para-Cresol | Verbenone | -3.834714636 | 0.000126 | 0.014079538 | * |
| beta-Ionone | Skatole | -4.837932718 | 1.31E-06 | 0.00016662 | *** |
| beta-Ionone | Undecane | -4.350161168 | 1.36E-05 | 0.001646054 | ** |
| para-Cymene | Skatole | -4.090708216 | 4.30E-05 | 0.005031678 | ** |
| para-Cymene | Undecane | -3.602936665 | 0.000315 | 0.034925286 | * |
| Sabinene | Skatole | -4.858688955 | 1.18E-06 | 0.000151252 | *** |
| Sabinene | Undecane | -4.370917404 | 1.24E-05 | 0.001509452 | ** |
| 6-me-5-hepten-2-one | Skatole | -3.957522367 | 7.57E-05 | 0.008633357 | ** |
| 2-Nonanone | Eucalyptol | 4.086661336 | 4.38E-05 | 0.006476853 | ** |
| beta-Caryophyllene | beta-Ionone | 4.293081518 | 1.76E-05 | 0.002079281 | ** |
| beta-Caryophyllene | Eucalyptol | 5.486565099 | 4.10E-08 | 5.49E-06 | **** |
| beta-Caryophyllene | Nonanal | 4.701287497 | 2.59E-06 | 0.000323158 | *** |
| beta-Caryophyllene | para-Cresol | 4.93652484 | 7.95E-07 | 0.000103385 | *** |
| beta-Caryophyllene | para-Cymene | 3.545857016 | 0.000391 | 0.042655895 | * |
| beta-Caryophyllene | Sabinene | 4.313837755 | 1.60E-05 | 0.001909292 | ** |
| Camphene | Eucalyptol | 3.853741186 | 0.000116 | 0.013144901 | * |
| gamma-Terpinene | para-Cresol | 3.51991172 | 0.000432 | 0.046622569 | * |

*Typhaeus typhoeus*, Kruskal-Wallis, *P* < 0.001, Statistic: 146.916, N = 10

| **Volatile 1** | **Volatile 2** | **Statistic** | **P-value** | **P-value adjusted** | **Significance level** |
| --- | --- | --- | --- | --- | --- |
| 2-heptanone | alpha-Pinene | -5.24109697 | 1.60E-07 | 2.62E-05 | **** |
| 2-heptanone | beta-Caryophyllene | -5.645822736 | 1.64E-08 | 2.79E-06 | **** |
| 2-heptanone | Camphene | -3.567281368 | 0.000361 | 0.045448718 | * |
| 2-heptanone | Camphor | -5.426172571 | 5.76E-08 | 9.73E-06 | **** |
| 2-heptanone | Indole | -5.084494539 | 3.69E-07 | 5.97E-05 | **** |
| 2-heptanone | Sabinene | -5.12923809 | 2.91E-07 | 4.74E-05 | **** |
| 2-heptanone | Skatole | -6.170542572 | 6.81E-10 | 1.16E-07 | **** |
| 2-heptanone | Undecane | -5.403800796 | 6.52E-08 | 1.09E-05 | **** |
| 2-heptanone | Verbenone | -4.948230085 | 7.49E-07 | 0.000120575 | *** |
| 6-me-5-hepten-2-one | alpha-Pinene | -4.493110409 | 7.02E-06 | 0.000849303 | *** |
| 6-me-5-hepten-2-one | beta-Caryophyllene | -4.945375948 | 7.60E-07 | 0.000100316 | *** |
| 6-me-5-hepten-2-one | Camphor | -4.713561451 | 2.43E-06 | 0.000306715 | *** |
| 6-me-5-hepten-2-one | Indole | -4.31811319 | 1.57E-05 | 0.001825477 | ** |
| 6-me-5-hepten-2-one | Sabinene | -4.395384689 | 1.11E-05 | 0.001315863 | ** |
| 6-me-5-hepten-2-one | Skatole | -5.531730266 | 3.17E-08 | 4.28E-06 | **** |
| 6-me-5-hepten-2-one | Undecane | -4.688561848 | 2.75E-06 | 0.000343915 | *** |
| 6-me-5-hepten-2-one | Verbenone | -4.17947903 | 2.92E-05 | 0.003243172 | ** |
| Acetophenone | alpha-Pinene | -3.747667711 | 0.000178 | 0.018384109 | * |
| Acetophenone | beta-Caryophyllene | -4.19993325 | 2.67E-05 | 0.002990329 | ** |
| Acetophenone | Camphor | -3.968118753 | 7.24E-05 | 0.007751319 | ** |
| Acetophenone | Indole | -3.572670492 | 0.000353 | 0.034982569 | * |
| Acetophenone | Sabinene | -3.649941991 | 0.000262 | 0.026492253 | * |
| Acetophenone | Skatole | -4.786287568 | 1.70E-06 | 0.000220863 | *** |
| Acetophenone | Undecane | -3.94311915 | 8.04E-05 | 0.008525439 | ** |
| Nonanal | Sabinene | -4.431747748 | 9.35E-06 | 0.001121668 | ** |
| Nonanal | Skatole | -5.568093324 | 2.58E-08 | 3.50E-06 | **** |
| Nonanal | Undecane | -4.724924906 | 2.30E-06 | 0.000294657 | *** |
| Nonanal | Verbenone | -4.215842088 | 2.49E-05 | 0.002836866 | ** |
| para-Cresol | Sabinene | -4.286295514 | 1.82E-05 | 0.002089289 | ** |
| para-Cresol | Skatole | -5.42264109 | 5.87E-08 | 7.87E-06 | **** |
| para-Cresol | Undecane | -4.579472673 | 4.66E-06 | 0.000573364 | *** |
| para-Cresol | Verbenone | -4.070389854 | 4.69E-05 | 0.005068929 | ** |
| para-Cymene | Sabinene | -3.584033948 | 0.000338 | 0.033832808 | * |
| para-Cymene | Skatole | -4.720379524 | 2.35E-06 | 0.000298964 | *** |
| para-Cymene | Undecane | -3.877211106 | 0.000106 | 0.01098871 | * |
| beta-Ionone | Skatole | -4.08175331 | 4.47E-05 | 0.004871998 | ** |
| 2-Nonanone | Skatole | -3.957770549 | 7.57E-05 | 0.010213098 | * |
| alpha-Pinene | Nonanal | 4.529473467 | 5.91E-06 | 0.000721396 | *** |
| alpha-Pinene | para-Cresol | 4.384021234 | 1.17E-05 | 0.001374799 | ** |
| alpha-Pinene | para-Cymene | 3.681759667 | 0.000232 | 0.02362623 | * |
| beta-Caryophyllene | beta-Ionone | 3.495398993 | 0.000473 | 0.045441943 | * |
| beta-Caryophyllene | Nonanal | 4.981739007 | 6.30E-07 | 8.38E-05 | **** |
| beta-Caryophyllene | para-Cresol | 4.836286773 | 1.32E-06 | 0.000173296 | *** |
| beta-Caryophyllene | para-Cymene | 4.134025207 | 3.56E-05 | 0.003921108 | ** |
| Camphor | Nonanal | 4.749924509 | 2.03E-06 | 0.000262505 | *** |
| Camphor | para-Cresol | 4.604472275 | 4.14E-06 | 0.000512756 | *** |
| Camphor | para-Cymene | 3.902210709 | 9.53E-05 | 0.010008403 | * |
| gamma-Terpinene | Skatole | -3.504489757 | 0.000457 | 0.044375867 | * |
| Indole | Nonanal | 4.354476249 | 1.33E-05 | 0.00156061 | ** |
| Indole | para-Cresol | 4.209024015 | 2.56E-05 | 0.00289818 | ** |
| Indole | para-Cymene | 3.506762449 | 0.000454 | 0.044375867 | * |

*Thorectes valencianus*, Kruskal-Wallis, *P* < 0.001, Statistic: 153.006, N = 12

| **Volatile 1** | **Volatile 2** | **Statistic** | **P-value** | **P-value adjusted** | **Significance level** |
| --- | --- | --- | --- | --- | --- |
| Acetophenone | alpha-Pinene | -4.260786861 | 2.04E-05 | 0.002322276 | ** |
| Acetophenone | beta-Caryophyllene | -5.223959865 | 1.75E-07 | 2.24E-05 | **** |
| Acetophenone | Camphene | -4.800302081 | 1.58E-06 | 0.000198033 | *** |
| Acetophenone | Camphor | -3.793898691 | 0.000148 | 0.015274921 | * |
| Acetophenone | Indole | -4.402582528 | 1.07E-05 | 0.001240851 | ** |
| Acetophenone | Skatole | -5.239522804 | 1.61E-07 | 2.08E-05 | **** |
| Acetophenone | Undecane | -4.703466016 | 2.56E-06 | 0.000314611 | *** |
| 2-heptanone | alpha-Pinene | -3.890422728 | 0.0001 | 0.013309275 | * |
| 2-heptanone | beta-Caryophyllene | -4.854517143 | 1.21E-06 | 0.000191882 | *** |
| 2-heptanone | Camphene | -4.45061884 | 8.56E-06 | 0.001267223 | ** |
| 2-heptanone | Indole | -4.023507992 | 5.73E-05 | 0.007855256 | ** |
| 2-heptanone | Skatole | -4.834399603 | 1.34E-06 | 0.000211006 | *** |
| 2-heptanone | Undecane | -4.326818595 | 1.51E-05 | 0.002193538 | ** |
| 6-me-5-hepten-2-one | beta-Caryophyllene | -3.985841605 | 6.72E-05 | 0.007194825 | ** |
| 6-me-5-hepten-2-one | Camphene | -3.562183821 | 0.000368 | 0.034939358 | * |
| 6-me-5-hepten-2-one | Skatole | -4.001404544 | 6.30E-05 | 0.0068005 | ** |
| 6-me-5-hepten-2-one | Undecane | -3.465347756 | 0.00053 | 0.049247802 | * |
| beta-Ionone | Camphene | -4.17778452 | 2.94E-05 | 0.003292346 | ** |
| beta-Ionone | Indole | -3.780064967 | 0.000157 | 0.015835532 | * |
| beta-Ionone | Skatole | -4.617005243 | 3.89E-06 | 0.000471074 | *** |
| beta-Ionone | Undecane | -4.080948455 | 4.49E-05 | 0.004933753 | ** |
| Nonanal | Skatole | -6.104130528 | 1.03E-09 | 1.41E-07 | **** |
| Nonanal | Undecane | -5.568073739 | 2.58E-08 | 3.43E-06 | **** |
| para-Cymene | Skatole | -4.191618243 | 2.77E-05 | 0.003129781 | ** |
| para-Cymene | Undecane | -3.655561455 | 0.000257 | 0.025148733 | * |
| Sabinene | Skatole | -5.263731821 | 1.41E-07 | 1.85E-05 | **** |
| Sabinene | Undecane | -4.727675032 | 2.27E-06 | 0.000281611 | *** |
| para-Cresol | Skatole | -3.783523398 | 0.000155 | 0.015771626 | * |
| 2-Nonanone | Acetophenone | 3.549972051 | 0.000385 | 0.046617918 | * |
| 2-Nonanone | Nonanal | 4.413478767 | 1.02E-05 | 0.001495324 | ** |
| 2-Nonanone | Sabinene | 3.568542088 | 0.000359 | 0.04379473 | * |
| alpha-Pinene | beta-Ionone | 3.6382693 | 0.000274 | 0.026349729 | * |
| alpha-Pinene | Nonanal | 5.125394585 | 2.97E-07 | 3.77E-05 | **** |
| alpha-Pinene | Sabinene | 4.284995878 | 1.83E-05 | 0.00210154 | ** |
| alpha-Pinene | Verbenone | 3.503390496 | 0.000459 | 0.043181296 | * |
| beta-Caryophyllene | beta-Ionone | 4.601442304 | 4.20E-06 | 0.00050349 | *** |
| beta-Caryophyllene | Nonanal | 6.088567589 | 1.14E-09 | 1.54E-07 | **** |
| beta-Caryophyllene | para-Cresol | 3.767960459 | 0.000165 | 0.016458676 | * |
| beta-Caryophyllene | para-Cymene | 4.176055304 | 2.97E-05 | 0.003292346 | ** |
| beta-Caryophyllene | Sabinene | 5.248168882 | 1.54E-07 | 2.00E-05 | **** |
| beta-Caryophyllene | Verbenone | 4.4665635 | 7.95E-06 | 0.000937936 | *** |
| Camphene | Nonanal | 5.664909804 | 1.47E-08 | 1.97E-06 | **** |
| Camphene | para-Cymene | 3.75239752 | 0.000175 | 0.017339992 | * |
| Camphene | Sabinene | 4.824511097 | 1.40E-06 | 0.000176837 | *** |
| Camphene | Verbenone | 4.042905715 | 5.28E-05 | 0.005754421 | ** |
| Camphor | Nonanal | 4.658506414 | 3.19E-06 | 0.000388584 | *** |
| Camphor | Sabinene | 3.818107707 | 0.000134 | 0.013985838 | * |
| gamma-Terpinene | Nonanal | 3.906297695 | 9.37E-05 | 0.009840709 | ** |
| Indole | Nonanal | 5.267190251 | 1.39E-07 | 1.83E-05 | **** |
| Indole | Sabinene | 4.426791544 | 9.56E-06 | 0.001119047 | ** |
| Indole | Verbenone | 3.645186162 | 0.000267 | 0.025918236 | * |
| Skatole | Verbenone | 4.482126439 | 7.39E-06 | 0.000879445 | *** |
| Undecane | Verbenone | 3.94606965 | 7.94E-05 | 0.008421118 | ** |

The *post hoc* analyses are based on Dunn Tests with a Bonferroni adjustment of the P-values following a significant value from the Kruskal-Wallis Tests (*P* < 0.05). For *Anomius baeticus* and *Onthophagus maki* the Kruskal-Wallis *P*-values indicated that no significant difference in electrophysiological responses existed among the 19 tested compounds. All non-significant (N.S.) pairwise comparisons were removed to shorten the length of these tables. The asterisks denote the following significance values: **** *P* < 0.0001, *** *P* < 0.001, ** *P* < 0.01, * *P* < 0.05.
